# Metabolomics Signatures of Atherosclerosis in Cardiovascular Disease: A Narrative Systematic Review

**DOI:** 10.3390/jcm14228028

**Published:** 2025-11-12

**Authors:** Monica Pibiri, Antonio Noto, Antonio Dalu, Sandro Muntoni, Karolina Krystyna Kopeć, Martina Spada, Luigi Atzori, Cristina Piras

**Affiliations:** 1 Department of Biomedical Sciences, University of Cagliari, 09042 Cagliari, Italy; mpibiri@unica.it (M.P.); antoniodalu98@gmail.com (A.D.); smuntoni@unica.it (S.M.); latzori@unica.it (L.A.);; 2 Cardiovascular and Thoracic Department, Division of Cardiology, Città Della Salute e Della Scienza, 10126 Torino, Italy; 3 Department of Mechanical, Chemical and Materials Engineering, University of Cagliari, 09042 Cagliari, Italy; k.kopec@studenti.unica.it

**Keywords:** atherosclerosis, cardiovascular disease, metabolomics

## Abstract

**Background:** High-throughput metabolomics studies have promoted the discovery of candidate biomarkers linked to atherosclerosis (AS). This narrative systematic review summarises metabolomics studies conducted in (1) individuals with subclinical AS (assessed by imaging techniques such as carotid intimal media thickness, IMT, and coronary artery calcium, CAC), (2) patients with established atherosclerotic plaques, and (3) individuals with AS risk factors. **Methods:** The systematic search was conducted in the PubMed database according to the Preferred Reporting Items for Systematic Reviews and Meta-analysis (PRISMA) guidelines. The inclusion criteria were as follows: (i) publication date between 2009 and 2024; (ii) identification of potential biomarkers for AS in subjects with a diagnosis of AS or with one or more traits characteristic of the disease (i.e., CAC or IMT); (iii) identification of potential AS biomarkers in subjects with atherogenic clinical conditions (i.e., Down’s syndrome, DS, polycystic ovarian syndrome, PCOS, and systemic lupus erythematosus, SLE); (iv) metabolomic studies; and (iv) studies in human samples. Exclusion criteria comprised the following: (i) studies on lipid metabolic diseases unrelated to AS, (ii) “omics” results not derived from metabolomics, (iii) reviews and studies in animal models or cell cultures, and (iv) systematic reviews and meta-analyses. Of 90 eligible studies screened, 24 met the inclusion criteria. **Results:** Across subclinical and overt AS, consistent disturbances were observed in amino acid, lipid, and carbohydrate metabolism. Altered profiles included branched-chain amino acids (BCAAs), aromatic amino acids (AACs) and derivatives (e.g., kynurenine–tryptophan pathway), bile acids (BAs), androgenic steroids, short-chain fatty acids (FAs)/ketone intermediates (e.g., acetate, 3-hydroxybutyrate, 3-HB), and Krebs cycle intermediates (e.g., citrate). Several metabolites (e.g., glutamine, lactate, 3-HB, phosphatidylcholines, PCs/lysophosphatidylcholines, lyso-PCs) showed reproducible associations with vascular phenotypes (IMT/CAC) and/or clinical AS. **Conclusions:** The identification of low-weight metabolites altered in both subclinical and overt AS suggests their potential as candidate biomarkers for early AS diagnosis. Given the steady increase in deaths from cardiovascular disease, a manifestation of advanced AS, this finding could have significant clinical relevance.

## 1. Introduction

Atherosclerosis (AS) is the leading cause of death in both industrialized and developing countries. The World Health Organization (WHO) estimates that 17.9 million people die annually from cardiovascular disease (CVD), a clinical manifestation of advanced AS, representing ~32% of all deaths, with numbers projected to rise by 2030 [1]. Furthermore, despite progress in cardiovascular and pharmacological research, the WHO established that this number is set to rise to 24 million deaths globally each year by 2030 [2]. Indeed, although atherosclerotic CVD was once considered a problem concentrated in industrialized countries, we have experienced an “epidemiological transition”, and the disease now spans the globe [3]. Improved sanitation, immunization, and treatment of acute infections have reduced the prevalence of communicable diseases in developing countries, and more people are now surviving to experience chronic diseases such as AS [3]. The adoption of less healthy eating habits may also have contributed to this trend [3].

AS is a chronic, lipid-driven inflammatory disease of arterial walls characterized by progressive plaque formation, luminal narrowing, and potential plaque rupture/erosion with thrombosis [3]. As the lesion progresses, a gradual buildup of plaque or thickening and hardening of the inside wall of the arteries occurs, which reduces blood flow through the lumen and can lead to rupture or erosion of the blood vessels in the area of the lesion [4]. However, if a plaque suddenly ruptures, it can form a thrombus within the vessel that travels with the blood flow and leads to occlusion of the vessel at a specific site. The disease affects multiple vascular beds, and plaques that form at different sites have different clinical manifestations and pathological features [5]. The main clinical consequences include ischemic heart disease, ischemic stroke, and peripheral arterial disease (PAD) [5]. Several factors have been questioned as causal factors for the atherosclerotic process, including genetic and environmental factors. In humans, it takes several decades for clinical complications to develop. Among the many known risk factors involved in the development of AS are hypercholesterolemia, hypertension, diabetes, and smoking [2]. It is not clear what the causal factor associated with the onset of the atherosclerotic process is; however, it is generally believed that AS is a chronic vascular inflammation triggered by interactions between the aforementioned risk factors and the cells of the arterial wall.

Over the last 30 years, the molecular mechanisms underlying the pathogenesis of AS have been extensively studied using genetically modified animals, and lipid-lowering drugs to prevent and treat AS have been identified. However, despite these advances, questions remain about the pathogenesis of atherosclerosis, and there is a need to develop new animal models and novel therapeutics to treat patients who cannot be effectively treated with current drugs [1].

Due to the complexity and widespread invasiveness of AS, it is undoubtedly necessary to diagnose this pathology at a very early stage to implement primary preventive measures that can delay or halt the progression of the disease. Therefore, if the early pathological changes and the biomarkers of AS can be identified, this will play an important role in the prevention and treatment of AS and related diseases. The difficulty lies in selecting these biomarkers. The complexity of the mechanism of AS makes it impossible to successfully characterize its occurrence with “one particular gene” or “one particular protein” [2].

Metabolomics research plays a key role in identifying new therapeutic targets for diseases and discovering candidate biomarkers. Metabolomics is a technique that characterizes both physiological and pathological conditions using a spectrum of small-molecule metabolites in the human body [6]. Compared to the other omics technologies (i.e., genomics, transcriptomics, or proteomics), it focuses more on characterizing “what has happened” in the body. The advancement of this technology will allow for a more accurate characterization of complex conditions of multiple comorbidities based on characteristic changes in a range of metabolite profiles, thereby enabling earlier detection of disease onset and appropriate intervention measures. This technology has also been successfully and increasingly used in the study of AS and related diseases; however, conflicting results have been reported in different studies [7,8]. Therefore, this narrative systematic review aims to systematically investigate and integrate the various metabolic candidate biomarkers of AS in individuals with atherogenic clinical conditions (assessed by imaging techniques, such as carotid intimal media thickness, IMT, and coronary artery calcium, CAC) and in patients with established atherosclerotic plaques. This approach may help identify a panel of metabolites with potential utility as candidate biomarkers of subclinical AS across healthy and emerging-risk groups. The study could help refine potential therapies, support earlier prevention and treatment, reduce AS-related CV complications, and inform future drug development.

## 2. Materials and Methods

This systematic review followed the PRISMA guidelines and is reported in accordance with the PRISMA statement [9] (http://www.prisma-statement.org/, accessed on 8 March 2025) (Supplementary File S1).

### 2.1. Search Strategy

A systematic search was conducted in PubMed for metabolomics studies on AS and CVD published between January 2009 and December 2024 using the following combination of terms: “Metabolomics AND Atherosclerosis AND Cardiovascular Disease AND not Review”. The titles and abstracts of all identified studies were screened and reviewed using defined selection criteria. The search was carried out independently by two reviewers.

### 2.2. Selection Criteria

**Figure 1:**
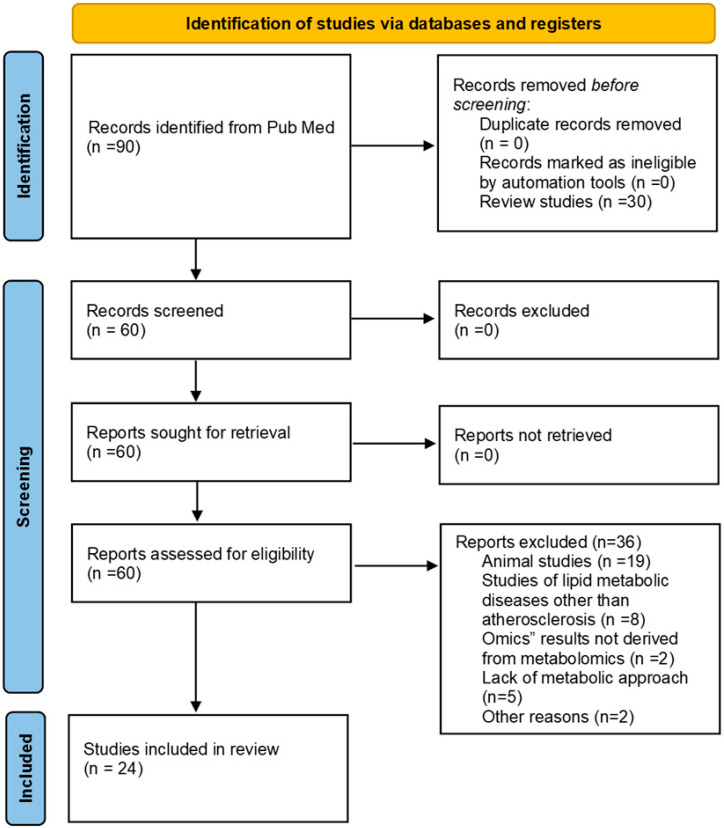
PRISMA flowchart. A systematic search of PubMed was conducted to identify metabolomics studies on AS and CVD published between January 2009 and December 2024, using the query “Metabolomics AND Atherosclerosis AND Cardiovascular Disease NOT Review.” Studies were included if they identified specific metabolites or potential biomarkers of AS in individuals diagnosed with AS or exhibiting related disease features or in subjects with atherogenic conditions such as Down’s syndrome, polycystic ovarian syndrome, or systemic lupus erythematosus. Only metabolomic studies based on human samples were considered. Studies on lipid metabolic diseases unrelated to AS, non-metabolomic “omics” analyses, reviews, systematic reviews, meta-analyses, and research conducted in animal models or cell cultures were excluded. The search was carried out independently by two reviewers.

English articles were selected for full-text review based on their titles and abstracts according to their relevance to the topic under investigation. The following inclusion criteria were applied without restriction to the biospecimens used: (i) identification of specific metabolites or potential biomarkers of AS in subjects with a diagnosis of AS or with a diagnosis of one or more features of the disease; (ii) identification of potential AS biomarkers in subjects with atherogenic clinical conditions (i.e., Down’s syndrome, DS, polycystic ovarian syndrome, PCOS, and systemic lupus erythematosus, SLE); (iii) metabolomic studies; and (iv) studies in human samples. In contrast, (i) studies on lipid metabolic diseases other than AS, (ii) “omics” results not derived from metabolomics, (iii) reviews and studies in animal models or cell cultures, and (iv) systematic reviews and meta-analyses were excluded from the review (Figure 1).

### 2.3. Data Extraction

The selected studies were thoroughly reviewed, and the following information was extracted from each article: first author, year of publication, sample size, baseline characteristics of participants, analysis platform used, use case, relevant candidate biomarkers, validation of biomarkers, statistical details, and relevant comments on the study (Table 1). Data extraction was conducted independently by two reviewers (Monica Pibiri and Antonio Dalu) with discrepancies resolved by discussion.

## 3. Results

The search process and selection of studies yielded a total of 90 original records, and screening of titles and abstracts excluded 30 unrelated records, including reviews. The full text of the remaining 60 articles was obtained. Of these, 36 unrelated records were excluded. Ultimately, a total of 24 studies were selected for this systematic review (Figure 1). Therefore, a narrative synthesis of the key findings was created (Table 1). At a second level, the results were organized and discussed according to the type of molecule identified (Table 2).

### 3.1. Study Characteristics

**Figure 2:**
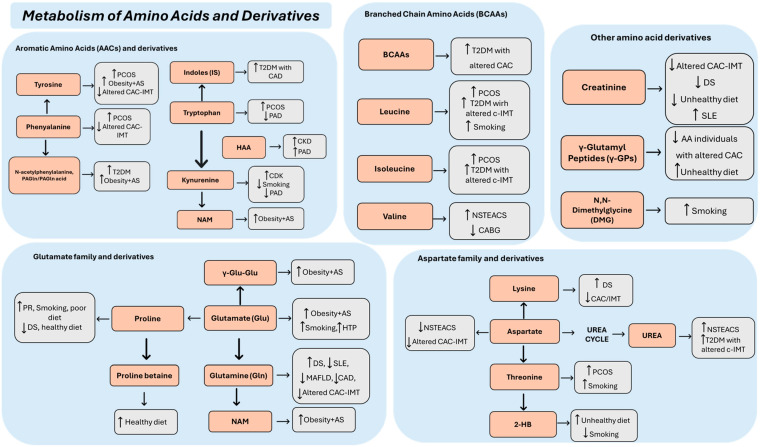
Summary of serum and plasma amino acids alterations across AS-related phenotypes. AS: atherosclerosis; NSTEACS: non-ST elevation acute coronary syndrome; PAD: peripheral arterial disease; CAD: coronary artery disease; T2DM: type 2 diabetes mellitus; BCAA: branched-chain amino acid; IMT: intima-media thickness; c-IMT: carotid intima-media thickness; PCOS: polycystic ovary syndrome; AACs: aromatic amino acid; CAC: coronary artery calcification; AAs: African Americans; CKD: chronic kidney disease; HTP: heated tobacco products; DS: Down’s syndrome; SLE: systemic lupus erythematosus; γ-Glu-Glu: γ-L-glutamil-L-glutamic acid; NAM: nicotinate D-ribonucleotide; MAFLD: metabolic-disfunction-associated fatty liver disease; HAA: 3-hydroxyanthranilic acid; 2-HB: 2-hydroxybutirate; CABG: coronary artery bypass grafting; ↑/↓: increased/decreased metabolite concentration.

**Figure 3:**
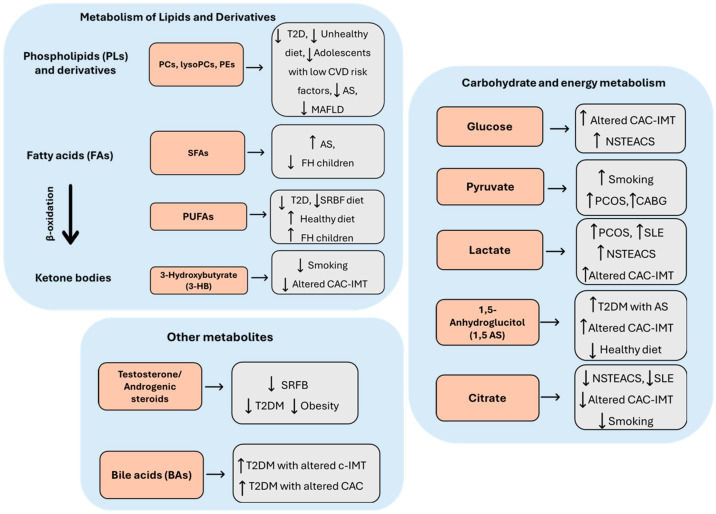
Summary of serum and plasma alterations in metabolites associated with carbohydrate and energy metabolism, lipid metabolism, BA metabolism, and androgenic steroid metabolism across AS-related phenotype. AS: atherosclerosis; NSTEACS: non-ST elevation acute coronary syndrome; FA: fatty acid; PUFAs: polyunsaturated fatty acids; SRFB: sugar-rich food and beverages; 1,5 AS: 1,5-anhydrosorbitol; PC: phosphocholine; FH: familial hypercholesterolemia; BAs: bile acids; SFAs: saturated fatty acids: CVD: cardiovascular disease; lysoPC: lysophosphatidylcholine; T2DM: type 2 diabetes mellitus; IMT: intima-media thickness; PCOS: polycystic ovary syndrome; 3-HB: 3-hydroxybutyrate; CAC: coronary artery calcification; CABG: coronary artery bypass grafting; PE: phosphatidylethanolamine; MAFLD: metabolic-disfunction-associated fatty liver disease; SLE: systemic lupus erythematosus. ↑/↓: increased/decreased metabolite concentration.

The 24 selected articles concerned a population aged between 10 and 84 years. In general, subjects with a diagnosis of AS or with one or more features of the disease were compared with healthy controls (Table 1). In particular, three studies were conducted with populations suffering from SLE [10], DS [11], and PCOS [12], i.e., diseases that are highly atherogenic but do not represent classic CV risk factors (Table 1). Plasma samples were used in 14 studies, serum in 10 studies, and urine in 1 study. In one study, two biological samples were used for the assessment of metabolites, namely, urine and plasma. Metabolites were mainly identified by targeted mass spectrometry (MS), gas chromatography–mass spectrometry (GC-MS), and liquid chromatography–mass spectrometry (LC-MS). In addition, nuclear magnetic resonance (NMR) spectroscopy was used in six studies. The identified metabolites are listed in Table 1 and Table 2, where they were classified by family and trend of variation as well as by the analytical method for metabolomics/lipidomics. The most important classes were amino acids and derivatives (Figure 2), carbohydrates and energy metabolites (Figure 3), lipids and derivatives (Figure 3), sex steroids, and BAs (Figure 3).

### 3.2. Metabolism of Amino Acids and Derivatives

As shown in Figure 2, metabolic profiling of patients with (subclinical) AS, assessed by CAC and/or IMT, two typical phenotypes of AS, showed reduced serum aspartate levels compared to controls [13]. Reduced aspartate levels were also found in plasma samples from patients with non-ST-elevation acute coronary syndrome (NSTEACS) compared to healthy controls and patients with stable AS [14]. The NSTEACS population also had higher urea levels compared to healthy controls [14], and the same trend was found in type 2 diabetes mellitus (T2DM) patients with altered carotid IMT (c-IMT) [15]. Contradictory results were found for creatinine, which was elevated in SLE patients [10] and decreased in individuals with DS [11], in individuals with altered CAC-IMT [13], and in those with an unhealthy diet [16]. Different trends were also observed for glutamate (Glu) and the glycine derivative N,N-dimethylglycine, which were increased in smokers [17] and decreased in patients with altered CAC-IMT with evidence of atherosclerotic plaques [13]. In addition, increased levels of the L-Glu derivative γ-L-glutamil-L-glutamic acid (γ-Glu-Glu) have been found to be associated with AS in obese patients [18]. Four studies have consistently found a decrease in glutamine (Gln) levels in populations at high atherogenic risk. In particular, decreased serum levels of this metabolite have been reported in patients with SLE [10], in individuals with altered IMT-CAC [13], and in obese patients with metabolic-dysfunction-associated fatty liver disease (MALFD) [19]. In addition, Santiago-Hernandez et al. [20] found low Gln levels in plasma and urine of patients undergoing coronary artery bypass grafting (CABG). Conversely, elevated serum Gln levels were found in DS patients [11]. Four studies were consistent in finding elevated plasma levels of branched-chain amino acids (BCAAs). BCAA metabolism was positively associated with CAC in the T2D patients analyzed by Chevli et al. [21]. In addition, increased plasma levels of leucine and isoleucine were found in cigarette smokers [17], PCOS women [12], and T2DM patients with altered c-IMT [22]. The latter population, as well as NSTEACS patients [14], also showed increased valine levels [22], while plasma valine levels were decreased in subjects undergoing CABG [20]. Lysine showed contradictory results in two studies, resulting in increased plasma levels in subjects with DS [11] and decreased levels in individuals with altered IMT-CAC [13]. Individuals with smoking habits [17] showed increased plasma levels of proline, while decreased levels of the metabolite were observed in DS patients [11] and in individuals who had a healthy diet [23]. Increased levels of proline betaine were found in two different studies by Gadgil et al. [23,24] in people with a healthy diet. Increased plasma levels of threonine were observed in patients with PCOS [12] and in smokers [17]. In addition, smokers [17] and women with PCOS [12], as well as obese patients with AS [18], showed increased tyrosine levels, which were decreased in individuals with pathologically altered CAC-IMT [13]. In addition, obese patients with AS [18], PCOS women [12], and European–American individuals (EAs) with T2DM [21] had higher plasma levels of phenylalanine or phenylalanine-derived products compared to control groups, while serum levels of this metabolite were decreased in individuals with altered CAC-IMT [13]. Kynurenine was increased in the plasma of patients with chronic kidney disease (CKD), who are at high risk for CV events [25], while it was decreased in smokers [17] and among metabolites associated with the microbiota of patients with peripheral artery disease of the lower extremities (PAD), a form of AS [26]. The latter population, together with CDK patients who are at high risk of CVD [25], also showed decreased tryptophan levels, while it was increased in the serum of PCOS patients [12]. In addition, elevated levels of 3-hydroxyanthranilic acid (HAA), a downstream product of tryptophan metabolism to kynurenine, were found in both CDK [25] and PAD patients [26], while elevated levels of the kynurenine metabolite nicotinate D-ribonucleotide (NAM) were associated with AS in obese patients [18]. The tryptophan derivative indoxyl sulphate (IS) increased in patients with PAD [26] and in patients with T2DM [15]. On the other hand, increased levels of γ-glutamyl dipeptides (γ-GPs) were found in the plasma of individuals with unhealthy diets [16], and a decrease was observed in African American individuals (AAs) with altered CAC [21]. Finally, 2-hydroxybutirate (2-HB) was increased in the plasma of individuals with unhealthy diets [16] and decreased in the plasma of smokers [17].

### 3.3. Carbohydrate and Energy Metabolism

As shown in Figure 3, both the populations with altered CAC-IMT examined in the study by Tzoulaki et al. [13] and the population of subjects with NSTEACS examined by Vallejo et al. [14] showed increased serum or plasma glucose levels, respectively, compared to healthy controls. Elevated pyruvate levels were found in the plasma of individuals with smoking habits, of women with PCOS [12], and of patients with acute plaque events after CABG [20]. Three studies found a different trend for 1,5-anhydrosorbitol (1,5 AS), which was increased in individuals with pathologic alterations in CAC-IMT and in T2DM patients with altered c-IMT [15] and decreased in patients who had a healthy diet [23]. Four studies have shown a change in lactate concentrations in populations at atherogenic risk. In particular, elevated levels of this metabolite were observed in the serum of individuals with SLE [10], of PCOS patients at high atherogenic risk [12], and of individuals with altered CAC-IMT [13], as well as in the plasma of patients with NSTEACS [14]. Finally, three studies have shown decreased citrate levels in patients at CV risk, such as individuals with altered CAC-IMT, smokers [17], and patients with NSTEACS [14]. In contrast, an age-related increase in citrate concentration was observed in SLE patients [10].

### 3.4. Metabolism of Lipids and Derivatives

As shown in Figure 3, alterations in phospholipid (PL) metabolism have been associated with the development of CVD. Specifically, decreased levels of phosphatidylcholine (PC) and/or lyso-phosphatidylcholine (lyso PC) were found by Paapstel K et al. [27] in AS patients, by Chevli et al. [21] in T2D patients, and by Gadgil et al. [24] in individuals on unhealthy diets. In addition, decreased levels of PC (18:2/20:2) and phosphatidylethanolamine (PE) (20:2/16:0) were associated with carotid AS (CAS) in both non-obese and obese patients with metabolic-associated fatty liver disease (MAFLD), while decreased levels of phosphatidylglycerol (PG) (18:0/20:4) were associated with CAS only in non-obese patients with MAFLD [19]. In addition, Syme et al. [28] found that PC16:0/2:0 was negatively associated with CVD risk factors in familial hypercholesterolemia (FH) adolescent patients, whereas a positive correlation was observed for PC14:1/0:0. Regarding saturated fatty acids (SFA), decreased SFA levels were found in children with FH compared to healthy children [29]. FH children also shared reduced acetate levels with patients with DS [11]. In addition, increased palmitate levels were found in patients with stable AS [30]. Smokers shared decreased plasma levels of the fatty acid (FA) oxidation product 3-hydroxybutirate with altered IMT-CAC individuals [13,17]. The FH children showed increased polyunsaturated fatty acid (PUFA) levels [29], whereas Chevli et al. [21] and Zheng et al. [16] observed an opposite result in AAs with pathologic alterations in CAC and in patients with unhealthy diets, respectively. The inverse correlation between unhealthy diet and unsaturated fatty acids (UFAs) was strengthened by Gadgil et al. [23], who found increased UFA levels in subjects with a healthy diet.

### 3.5. Other Metabolites

Androgenic steroids were found to decrease in the plasma of EA and AA T2D patients with altered CAC [21], in the serum of patients eating an unhealthy diet of sugar-rich foods and beverages (SRFB) [16], and in obese subjects at high CV risk [18] (Figure 3).

Moreover, increased bile acid (BA) concentrations were found in T2DM patients with altered c-IMT [31] or CAC [21] (Figure 3).

## 4. Discussion

This review highlights recurrent metabolite signatures across subclinical and overt AS, underscoring opportunities for earlier detection and mechanistic insight. The identification of these candidate biomarkers could revolutionize the management of the disease, starting with the possibility of early diagnosis, allowing patients to have a better prognosis, and avoiding the development of plaque to the most unfortunate acute events. Biomarkers may also be useful in identifying and evaluating the stages associated with disease progression and in monitoring the effectiveness of therapeutic treatments. In addition, the identification of a metabolomic profile associated with AS predisposition could allow for the early identification of CV risk groups and a targeted therapeutic approach. In this systematic review, 24 papers that met specific criteria were analyzed to identify common metabolites in patients at high CV risk—with or without overt atherosclerotic plaques—that could serve as potential biomarkers. To this end, the discussion will focus on the metabolites that show consistent support across multiple studies.

### 4.1. Metabolism of Amino Acids and Derivatives

#### 4.1.1. Aromatic Amino Acids (AACs) and Derivatives

Four studies here examined reported altered levels of phenylalanine or phenylalanine-derived metabolites in populations at CV risk (Figure 2). Phenylalanine is an essential AAC and the precursor for tyrosine and dopamine-related neurotransmitters. Phenylalanine and tyrosine have been shown to be associated with incident heart failure [34] and to increase the risk of T2D by impairing both insulin secretion and insulin sensitivity [35]. Furthermore, in a recent study of Jauhiainen et al. [36], phenylalanine was the only amino acid significantly associated with coronary artery disease (CAD), ischemic stroke, and CVD events, confirming the results of previous studies [37]. Interestingly, phenylalanine-derived metabolites produced by gut microbiota (GM) have also been found associated with the risk of CV events [38]. In particular, dietary phenylalanine is metabolized to phenylacetic acid by GM and converted into phenylacetylglutamine (PAGln) by the host. PAGln has been found to increase thrombosis, potentially via adrenergic receptors, and to be associated with increased major adverse CV events (MACE) [39]. Furthermore, a recent study of Heianza et al. [40] has shown that higher PAGln was associated with a higher risk of coronary heart disease (CHD), particularly in women with a diet high in animal proteins and poor in vegetable proteins. Therefore, adherence to vegetable-based diets was suggested to attenuate unfavorable associations between PAGln and CHD risk. Consistently, altered levels of phenylalanine or its derivatives have been found in the populations at CV risk here examined (Table 2, Figure 2). In particular, elevated phenylalanine and tyrosine levels were found in women with PCOS [12]. Increased tyrosine levels were also found in smokers [17] and in obese patients with AS [18]. In addition, increased levels of the phenylalanine derivatives N-acetylphenylalanine and PAGln/PAGln acid were found in EA individuals with T2DM [21] and in obese patients with AS [18], respectively. In contrast, Tzoulaki et al. [13] found decreased tyrosine and phenylalanine levels in individuals with altered CAC-IMT. Interestingly, in a previous study by Würtz et al. [37], phenylalanine was more strongly associated with CVD before the age of 60. Based on these results, we must therefore consider that the analysis of phenylalanine levels in the studies by Tzoulaki et al. [13], involving individuals significantly older than the participants in the study by Buszewska-Forajta et al. [12], may have been affected by the age of the participants.

Among the group of AACs, there is tryptophan. Elevated tryptophan levels were found in the serum of PCOS patients [12] (Table 2, Figure 2), possibly due to a disruption of the TCA cycle as a result of insulin resistance [41]. In contrast, the reduced tryptophan levels observed in patients with PAD [26] and in CDK patients at high CV risk [25] (Table 2, Figure 2) may be related to inflammation, which plays an important role in CDK and in all stages of AS [42]. During inflammation, pro-inflammatory cytokines increase tryptophan degradation by promoting the expression of the enzyme indoleamine dioxygenase (IDO) in macrophages and other cells [43]. Consistently, a link between IDO activity and AS development has been demonstrated [43,44,45,46]. Upregulated IDO activity increases the conversion of tryptophan to kynurenine, thus resulting in higher levels of the kynurenine–tryptophan ratio (KTR), considered a marker of cell-mediated immune activation [47]. The tryptophan–kynurenine pathway, particularly, elevates the KTR in CVD, linking IDO activity to atherosclerotic plaque formation [44,48,49]. Tryptophan degradation products also accumulate in CDK due to reduced renal excretion [50]. Consistent with these findings, increased kynurenine levels have been found in CDK patients at high CV risk in the study of Benitez et al. [25] here examined (Table 2, Figure 2). Opposite decreased kynurenine levels have been found in smokers [17] and in metabolites associated with the microbiota of patients with PAD [26] (Table 2, Figure 2). In a previous study, Ho et al. [26] found that tryptophan and the KTR positively correlated with advanced AS [51]. Although such a correlation was not observed in the study presented here by the same authors, a significant correlation was found between these metabolites and the development of MACE during the follow-up period. This was suggested to be related to the more pronounced baseline inflammation that occurs in patients with advanced AS and increased IDO1 activity [26]. Furthermore, increased HAA levels were found in both CDK and PAD patients [25,26] (Table 2, Figure 2). HAA is a downstream product of tryptophan metabolism to kynurenine. In a cohort of patients with stable angina, plasma HAA, in addition to other kynurenines, was associated with risk of acute myocardial infarction and correlated with metabolic syndrome phenotypes, suggesting that these metabolites could be used to improve risk estimates [52]. Consistently, increased levels of the kynurenine metabolite NAM were found to be associated with AS in obese patients by Menaker et al. [18] (Table 2, Figure 2).

Among the tryptophan derivatives, there are indole metabolites, IS and indole-3-aldehyde (I3A). IS is an important endogenous uremic toxin that is mainly produced by the absorption and accumulation of GM metabolites. There is growing evidence for the importance of the GM in AS, where gut-derived uremic toxins (GDUTs) may play an important rule. IS is a protein-bound toxin that binds mostly to albumin in the blood [53]. This binding form occurs more frequently in CDK patients when their kidney function deteriorates [54]. Albumin-bound IS forms not only impair its distribution and clearance efficiency in the body but can also exert toxic effects on the CV system via albumin-mediated pathways [54]. As the condition of CKD worsens, the accumulation of IS increases, and the proportion of its binding to albumin increases accordingly, which may further increase the risk of CV damage. IS not only leads to EC damage and promotes vascular SMC proliferation but is also associated with AS development, cardiac hypertrophy, and fibrin synthesis [52]. In AS patients, IS is elevated in plasma, possibly due to an increased ability to produce GDUTs, which exposes them to the harmful effects of these toxins [55]. Moreover, there are very few strategies for its elimination, and most of these show only an incomplete effect [56]. In line, in the study by Ho et al. [26], a significant positive correlation was found between IS levels, the microbiota, and the presence of AS (Table 1 and Table 2, Figure 2). In addition, a positive correlation was found by Omori et al. [15] between IS and max-IMT in T2DM patients who never experienced a CVD (Table 2, Figure 2). In addition, IS plasma levels were significantly higher in T2DM subjects who survived CAD [15] (Table 2, Figure 2). As suggested by the authors, the plasma IS elevation in diabetics may be related to the diabetes-mediated alterations to the GM composition and metabolism [57,58].

#### 4.1.2. Aspartate Family and Derivatives 

Decreased aspartate levels were found in the serum of individuals with altered CAC-IMT by Tzoulaki et al. [13] and in plasma samples from NSTEACS patients by Vallejo et al. [14] (Table 2, Figure 2). A recent study by Zhao et al. [59] has shown that aspartate may play a beneficial role in ischemic heart disease and blood pressure, although the mechanism is not yet clear. Therefore, the decreased aspartate levels found in the two populations studied by Tzoulaki et al. and Vallejo et al. could represent a CVD risk factor.

In plants and micro-organisms, the aspartate family pathway leads to four key essential amino acids for humans: lysine, methionine, threonine, and isoleucine [60]. Individuals with altered IMT-CAC [13] and DS patients [11] shared altered lysine levels but with an opposite trend (Table 2, Figure 2). The amino acid lysine is crucial for collagen synthesis and essential for the structure of arterial walls. Collagen is the most abundant protein in the ECM, and the telopeptide regions of collagen molecules contain lysine and hydroxylysine residues, which are responsible for the formation of cross-links through chemical bonds once the collagen molecules have self-assembled [61]. The copper-dependent aminooxidase enzyme lysyl oxidase (LOX) catalyzes the cross-linking of collagen and elastin to maintain the tensile strength and structural integrity of the vasculature. LOX supports cross-linking by oxidative deamination of the lysine and hydroxylysine residues, which then enable the formation of the cross-links [61]. Therefore, lysine deficiency could lead to weaker arterial walls that are prone to plaque formation. Consequently, Tzoulaki et al. [13] found reduced levels of the metabolite in individuals with altered IMT-CAC. On the other hand, Hetman et al. [11] found increased plasma levels of lysine in DS patients (Table 2, Figure 2). In this context, it is important to consider that excessive LOX activity increases vascular stiffness and the severity of occlusive disease [62]. Recently, Stoyell-Conti et al. [63] deciphered the mechanism behind the atherogenic and osteogenic nature of LOX in the vasculature and found that inactivation of LOX in smooth muscle cells (SMCs) protects hyperlipidemic mice from AS and plaque calcification by reducing SMC modulation and the pro-osteogenic FAK/β-catenin signalling axis [64,65]. This suggests that elevated lysine levels may favor the development of atherosclerotic plaques when accompanied by increased LOX activity. However, it is also important to remember that although changes in LOX gene expression have been reported in patients with T2DM, no correlation has been found between serum LOX levels and the development of CVD [66]. Therefore, to clarify the significance of elevated lysine levels in patients with DS, it would be important to evaluate LOX activity and a possible correlation with CV risk.

Increased plasma levels of the glucogenic amino acid threonine have been observed in patients with PCOS [12] and in smokers [17] (Table 2, Figure 2), possibly due to a reduced gluconeogenesis response already observed in women with PCOS [41,67] and in mice exposed to cigarette smoke [68,69]. Moreover, increased levels of the threonine catabolite 2-HB were found in the plasma of people who had an unhealthy diet [16], while they decreased in the plasma of smokers [17] (Table 2, Figure 2). 2-HB, the conjugate base of 2-hydroxybutyric acid, is formed in mammal tissues (mainly in the liver) that break down L-threonine or synthesize glutathione. This metabolite can serve as an early indicator of insulin resistance in non-diabetics [70] and as a predictor of a deterioration in glucose tolerance [71]. Interestingly, Deidda et al. observed decreased concentrations of 2-HB in the plasma of patients with evidence of myocardial ischemia with stenotic disease compared to individuals without evidence of ischemic heart disease, who constituted the control group [72]. Conversely, patients with evidence of myocardial ischemia without stenosis had elevated 2-HB levels compared to the control group. The demonstration of an opposing trend in 2-HB levels associated with different stages of myocardial ischemia (high metabolite levels preceding the onset of stenosis, in which metabolite levels decrease) [72] suggests that altered butyrate production in smokers [17] and in individuals with unhealthy diets [16] may be considered a CV risk condition that precedes the onset of marked coronary vascular changes.

Aspartate is also a metabolite in the urea cycle. Urea and other nitrogenous metabolic products are generated by amino acid catabolism. Normally, urea is excreted by the kidneys in the urine. If kidney function is impaired, there is a steady accumulation of urea in the blood. For a long time, the toxicity of urea was considered negligible [73]. However, a study in an animal model of chronic renal failure (CRF) and accelerated AS showed that plasma urea was the only significant predictor of aortic plaque area [74]. This suggests that the high urea levels in chronic dialysis patients with CDK may play an important role in accelerated AS in these patients [75]. The observation that oral administration of urea accelerated atherogenesis in non-uremic ApoE^−/−^/mice fed a high-fat diet supports this hypothesis [76]. Urea exerts its toxic action through ROS production [77,78]. Recently, a positive association between blood urea nitrogen (BUN), which accounts for the nitrogen component of urea, and CV mortality has been found in CDK patients undergoing hemodialysis [79]. In addition, higher BUN levels were significantly associated with the occurrence of heart failure in a community-based cohort [80]. Consistent with this evidence, high urea levels were found in two populations at high CV risk examined in this study, namely, NSTEACS patients [14] and T2DM patients with altered c-IMT [15] (Table 2, Figure 2).

#### 4.1.3. Glutamate Family and Derivatives

Several studies have shown that L-Glu-metabolism-derived metabolites, such as Gln and proline, are associated with cardiometabolic factors. Gln is classified as a conditionally essential amino acid and is an important substrate for many biosynthetic pathways involved in the regulation of cellular functions [81]. Gln is synthesized predominantly from Glu and ammonia (NH3) by the largely cytosolic enzyme Gln synthetase (GS), while the mitochondrial enzyme glutaminase (GLS) is responsible for the hydrolysis of Gln to Glu and NH3. Gln also functions as an L-arginine precursor for NO synthesis [82].

As discussed in detail by Durante et al. [81], several studies have shown an inverse correlation between Gln levels and the development of cardiometabolic disease, a constellation of metabolic disorders characterized by insulin resistance, impaired glucose tolerance, dyslipidemia, hypertension, and central obesity. Individuals with cardiometabolic diseases are prone to diabetes and subsequent CV events [83,84]. Aside from serving as a candidate biomarker for cardiometabolic disease, the Gln-cycling pathway may be a factor influencing metabolic risk. Indeed, beneficial effects of Gln supplementation have been observed in diabetics [85,86,87,88] and on some CV risk factors in animals exposed to exercise or a high-fat diet [89,90]. The beneficial effects of Gln on cardiometabolic risk may be mediated by a number of mechanisms, including enhanced release of glucagon-like peptide 1, externalization of glucose transporters, stimulation of insulin release by pancreatic cells, transcription of insulin-dependent genes, and improved insulin disposition [85,88,91]. On the other hand, the reduction in blood pressure by Gln may be due to the inhibition of arterial tone promoted by an increase in NO production as a result of an increase in L-arginine synthesis [82]. The release of NO by endothelial cells (ECs) not only regulates blood flow and blood pressure but also maintains blood fluidity and prevents thrombosis by limiting platelet aggregation and adhesion. In addition, NO also protects against intimal thickening by inhibiting SMC activity and attenuates the development of AS by blocking the inflammatory response within the vessel wall [92,93,94]. Therefore, dysfunction or loss of ECs is a known response to CV risk factors that precedes the development of AS and other CV disorders. Recent studies have shown that Gln plays a central role in promoting EC function and viability [95,96,97] and also protects ECs from various deleterious stimuli, including oxidative stress, hypertension, infection, and hyperglycemia [98,99,100,101]. Interestingly, Gln-derived ammonia (NH3), long considered a potentially toxic product of GLS1, was also recently identified as a novel signalling gas in the vasculature that promotes EC survival [102]. Overall, these results indicate that Gln plays a protective role against CVD due to its ability to counteract oxidative stress and inflammation. In line with this evidence, four of the studies reviewed here reported decreased Gln levels in individuals at CV risk, namely, patients with SLE [10], obese patients with MALFD [19], subjects with altered CAC-IMT [13], and patients with CAD undergoing CABG [20] (Table 2, Figure 2). Conversely, elevated serum Gln levels were found in DS patients by Hetman et al. [11] (Table 2, Figure 2). DS is a complex disease that causes deleterious effects in many organs, including the CV system [103]. Given the crucial role that Gln plays in protein synthesis and cellular energy, it has been hypothesized that the elevated levels of the metabolite are indicative of broader metabolic shifts potentially affecting vascular health and stress responses in DS patients [11]. On the other hand, a chronic inflammatory state and the increase in oxidative stress caused by mitochondrial dysfunction in cells and organs affected by trisomy 21 may represent the key points common to most DS phenotypes [104]. Therefore, given the antioxidant and anti-inflammatory effects of Gln, the increase in Gln levels may represent a protective mechanism to counteract the redox imbalance and inflammatory state in individuals with DS.

In contrast to the cardioprotective effect of elevated Gln levels, increased Glu levels have been found to be associated with increased CV risk. In particular, high plasma Glu levels were found to be associated with higher body mass index (BMI), blood pressure, and insulin resistance, whereas Gln levels, or the Gln:Glu ratio, were inversely associated with these parameters [105,106,107]. Consistently, in elderly patients at high CV risk, baseline levels of circulating Glu were found to be associated with increased risk of CV events, whereas the plasma Gln:Glu ratio was associated with decreased risk, particularly in relation to stroke [108]. In addition, Glu induces oxidative stress and apoptosis in cerebral ECs [109]. Consistently, the study by Harada et al. [17] included in this systematic review showed that cigarette smokers and HTP users had higher Glu levels than non-smokers (Table 2, Figure 2). Specifically, it has been shown that smoking is associated with the Glu signalling pathway, which can lead to endothelial dysfunction and AS in the vasculature. In addition, Menaker et al. [18] found that elevated levels of the Glu derivative γ-Glu-Glu were associated with AS in obese patients [18]. In contrast, Tzoulaki et al. [13] showed decreased Glu levels in individuals with altered CAC-IMT (Table 2, Figure 2). Since Glu is involved in both the synthesis and degradation of glutathione, its decrease has been linked to glutathione deficiency, triggering oxidative stress, a key factor in AS pathogenesis [13]. Notably, the potential mechanisms of Glu’s negative effects on cardiometabolic factors are still largely unclear and require further investigation.

Proline is a non-essential amino acid synthesized from glutamic acid that is involved in several biological activities, such as collagen synthesis, immune response, and energy production. In addition, several studies have shown that proline modulates the intracellular redox environment against oxidative stress [110,111], and there is also evidence that its degradation can increase ROS production [112]. On this basis, Luo et al. recently correlated proline levels with the presence of atherosclerotic plaques. Their results have shown that proline may serve as a non-invasive candidate biomarker to differentiate plaque erosion (PE) and plaque rupture (PR), the two major subtypes of ST-segment elevation myocardial infarction (STEMI), one of the most serious forms of CV disease. In particular, it has been suggested that the high proline levels found in PR patients serve as a substrate for increased ROS production leading to oxidative stress, one of the major mechanisms of PR. Moreover, since collagen fibers degraded by matrix metalloproteinases produce excess proline [112], the high proline levels in PR patients may indicate the degradation of collagen fibers in the plaque, leading to the formation of thin plaque fiber caps [113]. Consistent with these findings, the study of Harada et al. [17] discussed in this review showed an increase in proline levels associated with pro-atherogenic factors such as cigarette smoke [17] (Table 2, Figure 2). In addition, in line with previous studies [114], Gadgil et al. [23] observed lower proline levels in individuals who had a healthy diet, while poorer diet quality was associated with higher circulating proline concentrations (Table 2, Figure 2). Several studies have emphasized the association between a healthy diet and a lower incidence of cardiometabolic disease [115,116,117]. Of note, a recent study that examined the metabolic profile of different body shapes (categorized by BMI and waist-to-height ratio) and their association with CVD risk found that increased arginine and proline metabolism was a common feature of different obesity subtypes, which correlated with increased CV risk [118]. Decreased proline levels were also found in the serum of individuals with DS by Hetman et al. [11] (Table 2, Figure 2), possibly reflecting the increased ROS production that leads to the oxidative stress often observed in the tissues of individuals with DS [119].

Serum levels of the proline derivative proline betaine are higher in people with healthy diets [23,24] (Table 2, Figure 2). This xenobiotic, abundant in citrus fruits, is a marker of specific healthy dietary patterns [120,121,122]. In such contexts, proline betaine is inversely associated with blood pressure, likely via increased NO production [123,124,125]. Noerman et al. also found inverse associations between proline betaine levels and plasma lipid profile, BMI, fasting insulin, high-sensitivity C-reactive protein (hs-CRP), smoking, and alcohol intake, suggesting a possible dietary role in the prevention/treatment of CVD [126]. A recent study further confirmed the link of proline betaine with a healthy diet, showing that urinary proline betaine is among the most pronounced metabolomic changes after consumption of fruits and vegetables (urinary proline also increases but less so) [127]. Notably, a combined fruit- and vegetable-related metabolite score is inversely associated with systolic blood pressure and BMI, and adding proline betaine, N-methyl proline, or proline to the model further strengthened this relationship [127].

Overall, these studies suggest that Glu metabolites play an important role in the pathophysiology of CVD and may represent early candidate biomarkers for AS.

#### 4.1.4. BCAAs

Increased circulating concentrations of BCAAs have been found to be associated with insulin resistance, T2DM and CHD, and an altered microbiome [128,129,130,131,132]. Several studies examined in this systematic review have shown a positive correlation between the increase in BCAAs and known atherovascular risk parameters. In particular, BCAA metabolism was positively associated with CAC in T2D patients analyzed by Chevli et al. [21], while increased leucine and isoleucine levels were found in cigarette smokers [17], in T2DM patients with altered c-IMT [22], and in PCOS patients [12] (Table 2, Figure 2). In addition, increased valine levels have been found to be associated with acute plaque events [14] and with c-IMT in T2DM patients [22], while an inverse correlation has been found in CABG patients by Santiago-Hernandez et al. [20] (Table 2, Figure 2). BCAAs are critical for protein synthesis, energy production, and cell signalling. Several mechanisms may explain the fluctuations in circulating BCAA concentrations: excessive food intake, increased production by GM, increased protein degradation, and decreased BCAA degradation in muscle and adipose tissue by lower expression of BCAA degradation genes [105,128,130]. Recently, Zhang et al. found that a high-protein diet promoted atherogenesis in the AS-prone ApoE^−/−^mouse model via amino-acid-mediated mTORC1 signalling and subsequent impairment of autophagy and mitophagy in macrophages [133]. In addition, more recently, the same authors [134] have shown that increased dietary leucine intake was both necessary and sufficient to trigger the pro-atherogenic effect of high protein intake in vivo, further supporting the pro-atherogenic role of increased BCAAs.

#### 4.1.5. Other Amino Acid Derivatives

Creatinine is a breakdown product of the amino acid derivative creatine, which reflects changes in energy metabolism in the muscles. The serum creatinine/serum cystatin C ratio, called the sarcopenia index (SI), is used as a surrogate marker for sarcopenia [135,136,137,138]. Previous studies have shown an inverse correlation between SI and incident CVD events and mortality [139,140]. Recently, Hashimoto et al. [141] found that SI is associated with the prevalence of subclinical AS in T2DM patients, possibly explained by the increased ROS production affecting both AS plaque formation and sarcopenia-associated muscle hydrolysis and protein synthetic activity [142,143]. In accordance with the inverse correlation between creatinine levels and CV risk, decreased levels of the metabolite were found in DS patients, in individuals with altered CAC-IMT [13], and in individuals with an unhealthy diet [15] (Table 2, Figure 2). In addition, an age-related increase in creatinine levels was observed in SLE patients by Jury et al. [10] (Table 2, Figure 2), which was greatly influenced by hydroxychloroquine treatment, capable of reducing creatinine levels in younger patients.

Two studies examined here have shown altered levels of γ-GPs in populations at high CV risk. In particular, increased levels of γ-GPs were found in the plasma of individuals with unhealthy SRFB diets [16] while a decrease was observed in AA individuals with altered CAC [21] (Table 2, Figure 2). γ-GPs are a class of low-molecular-weight peptides containing γ-glutamyl residues and forming a γ-bond between the γ-carboxyl group of glutamic acid and the amino group of the subsequent amino acid [144]. These metabolites have been studied in terms of their multiple biological functions affecting human health, including anti-inflammatory [145,146], antioxidant [147], and hypoglycemic effects [148], among others. These physiological functions of γ-GPs are caused by the allosteric activation of the calcium-sensing receptor (CaSR5) [146,149], a cell surface G-protein-coupled receptor ubiquitously present in many mammalian tissues, including the vasculature [150]. Primarily, the CaSR plays an essential role in Ca^2+^ homeostasis [151]. However, recent studies have shown that CaSR can modulate a variety of cellular processes associated with inflammation and the CV system, such as AS, hypertension, vascular calcification, obesity, and myocardial infarction [150]. In particular, during AS progression, CaSR is expressed in various vascular cell types, as the atherosclerotic lesion is a good source of extracellular calcium. This attracts more immune cells, which invade the lesions, leading to plaque growth. Several studies have indicated that CaSR may play a protective role against AS and vascular calcification by modulating and inhibiting Ca^2+^ deposition in atherosclerotic plaques by SMCs. Based on these findings, the concentration of γ-GPs is expected to decrease in the presence of CV risk factors. Consistently, Chevli et al. [21] found reduced levels of γ-glutamyl valine, γ-glutamyl methionine, γ-glutamyl leucine, and γ-glutamyl-alpha-lysine in AA patients with altered CAC. In contrast, the increased levels of γ-GPs found by Zheng et al. [16] in individuals with unhealthy diets were suggested to be related to hyperglycemia-induced oxidative stress, leading to γ-GPs biosynthesis [152,153] (Table 2, Figure 2).

A different trend was observed in the studies here reviewed for the glycine derivative N,N-dimethylglycine (DMG), which increased in patients with smoking habits [17] and decreased in patients with altered CAC-IMT [13] (Table 2, Figure 2). DMG is a known feedback inhibitor of betaine homocysteine methyltransferase (BHMT), a zinc metalloenzyme that converts glycine betaine to DMG. On this basis, McGregor et al. [154] postulated that DMG accumulation through inhibition of BHMT activity may be responsible for hyperhomocysteinemia-induced AS in patients with CRF. In addition, Lind et al. [155] recently found that DMG was among the top five metabolites associated with future CVD as well as with subclinical markers of CVD, such as enlarged left-atrial diameter and decreased ejection fraction, in a population-based sample [155]. While the results of Harada et al. [17] are consistent with these findings, the inverse correlation found by Tzoulaki et al. [13] for both CAC-IMT and CAD requires further analysis.

### 4.2. Carbohydrate and Energy Metabolism

Energy metabolism, the Krebs cycle, and glycolysis are central metabolic pathways in the pathogenesis of AS [156]. Increased glycolysis, decreased FA oxidation flux, and increased amino acid anaplerosis have been shown to characterize high-risk rupture-prone atherosclerotic plaques associated with increased CV risk [156].

#### 4.2.1. Glucose, Pyruvate, and Lactate

Individuals with a combination of central and global obesity, known CVD risk traits, have elevated plasma glucose levels, possibly due to delayed glucose catabolism [157]. In line, increased glucose levels have been found in patients with altered CAC-IMT by Tzoulaki et al. [13] and in patients with acute plaque events by Vallejo et al. [14] (Table 2, Figure 3). In addition, the glycolysis product pyruvate was found to be increased in the plasma of smokers [17] and in PCOS women [12] as well as in the urine of CAD patients undergoing CABG [20] (Table 2, Figure 3). During anaerobic glycolysis, pyruvate is converted into lactate. Previously considered merely a by-product of glycolysis, lactate has been recently redefined as a molecule with far-reaching physiological and pathological effects. It is now known that lactate serves as the primary carbon source for the TCA cycle in all tissues except the brain and even outperforms glucose [158]. In inflamed tissues, such as atherosclerotic plaques, lactate concentrations rise sharply [159].

Since lactate production is an indicator of inflammation and local hypoxia can occur in highly active inflamed tissues, such as atherosclerotic plaques [160], it has been hypothesized that hypoxia is the factor responsible for pro-atherosclerotic processes, such as deficient lipid efflux, inflammation, interference with macrophage polarization, and glucose metabolism [161]. However, recently, Li et al. have proposed a new mechanism by which lactate is involved in vascular inflammation during AS [162]. According to the authors’ findings, lactate uptake via monocarboxylate transporter 1 (Mct1) in ECs within atherosclerotic plaques triggers an inflammatory response in these cells that requires the conversion of lactate to pyruvate as well as the production of NADH and oligomerization of the NADH-sensitive transcriptional corepressor C-terminal binding protein 1 (CtBP1). In line with this evidence, Vallejo et al. [14] observed elevated lactate levels in patients with NSTEACS, and Tzoulaki et al. [13] found a strong correlation between elevated lactate levels and plaque burden in AS patients independent of traditional CV risk factors (Table 2, Figure 3). In addition, high lactate levels have been found in patients with diseases that are associated with high CVD risk, such as SLE [10] and PCOS [12] (Table 2, Figure 3).

#### 4.2.2. 1,5-AS

Altered 1,5-AS levels were detected in two of the studies discussed here. 1,5-AS, is a marker of short-term glycemic control that inversely correlates with glucose concentration. Physiologically, 1,5-AS is present in high-but-constant concentrations in the blood. It is freely filtered by glomeruli and reabsorbed in renal tubules, with a small amount corresponding to food intake being excreted in urine. In hyperglycemia, high levels of glucose block renal tubular reabsorption of 1,5-AS, resulting in a decrease in its serum concentration. Therefore, low serum 1,5-AS may serve as a marker of short-term hyperglycemia, and concentrations are thought to reflect hyperglycemic episodes over a period of 1–2 weeks [163,164]. Lower 1,5-AS levels have been associated with an increase in serious CV events in normoglycemic patients [165]; however, there is a stronger association in diabetics [166]. A recent study by Li et al. [167] conducted in T2DM patients highlighted the association between 1,5-AS levels, AS, and diabetes. Specifically, higher intracranial plaque enhancement was shown in a group of patients with higher short-term glycemic variability, which was characterized by lower 1,5-AS levels, than in a group with low short-term glycemic variability with higher 1,5-AS levels. In addition, Sanakara et al. found a positive correlation between plasma and salivary 1,5-AS levels and IMT in T2DM patients [168]. In contrast, in the study by Omori et al. [15], elevated 1,5-AS levels were found in T2DM patients with subclinical AS (altered maximal IMT), and this metabolite was also significantly associated with CAD (Table 2, Figure 3). Accordingly, Tzoulaki et al. [13] found increased 1,5-AS levels in normoglycemic patients with pathologic changes in CAC-IMT (Table 2, Figure 3). In addition, according to previous results [169,170] in the study by Gadgil et al. [23], serum 1,5-AS levels were decreased in individuals without known CVD who had a healthy diet low in SFAs and rich in dairy products (Table 2, Figure 3). Overall, this evidence suggests a positive correlation between 1,5-AS levels and CV risk factors. However, it should be noted that previous clinical studies have not found a significant association between plasma 1,5-AS and carotid AS in subjects from the general population [171] or in patients with T2D or hypertension [172]. These results suggest that 1,5-AS is probably a marker of glycemic status rather than AS. However, it cannot be ruled out that measuring 1,5-AS levels in conjunction with other parameters may provide useful indications of CV risk, as found in the study by Sanakara et al. [168], where the combination of salivary 1,5-AS with salivary allantoin provided a better prediction of CV risk in patients with T2D.

#### 4.2.3. Citrate

Four studies analyzed in this review reported altered citrate levels in patients at CV risk. In particular, decreased levels of this metabolite were found in the serum of patients with altered CAC-IMT [13] and in the plasma of smokers [17] and of NSTEACS patients [14] (Table 2, Figure 3). Citrate is an intermediate product in the Krebs cycle and is thus of central importance for anaerobic energy metabolism, which plays a prominent role in the AS development. Recently, Tong et al. [2] have shown that AS patients have a lower concentration of citric acid than controls, which has been related to an apparent disturbance of whole-body energy metabolism and dysregulation of the Krebs cycle. Indeed, atherosclerotic plaques are rich in hypoxic regions, where limited oxygen availability leads to Krebs cycle dysregulation [161]. On the other hand, in SLE patients, citrate was among the metabolites whose plasma levels were positively associated with patient age but not with SLE disease activity [10] (Table 2, Figure 3). However, in the same study, citrate and creatinine were significantly reduced in patients treated with hydroxychloroquine. Since the proportion of younger patients in the study treated with hydroxychloroquine was higher than that of older patients, according to the authors, this could explain the age-related increase in these metabolites. Furthermore, a disease-wide association analysis showed that SLE-treatment-associated metabolites were significantly positively associated with the incidence of T1D and T2D but not with SLE itself. Therefore, these glycolytic metabolites could serve as candidate biomarkers of adverse treatment effects, helping to prevent comorbidities and improve patients’ quality of life.

### 4.3. Metabolism of Lipids and Derivatives

#### 4.3.1. PLs and Derivatives

Alterations in PL metabolism have been linked to CVD development, albeit with conflicting results. Circulating phospholipid patterns have been shown to be associated with metabolic risk factors, hepatic steatosis, and severity of inflammation in MAFLD. In particular, in the study of Shao et al. [19] here examined, decreased levels of the glycerophospholipids PC (18:2/20:2) and PE (20:2/16:0) were found to be associated with CAS in both non-obese and obese MAFLD patients, whereas decreased PG (18:0/20:4) was independently associated with CAS only in non-obese MAFLD patients (Table 2, Figure 3). Accordingly, Waś et al. recently reported a marked increase in PCs and PGs in patients at high cardiovascular risk (HCVR), emphasizing their potential as candidate biomarkers for early assessment of CV risk [173]. In addition, in the study of Shao et al. [19] lyso-PC (lyso-PC C18:0, lyso-PC C17:0) and PC (PC aa C36:3) were shown to have a potential role in the pathogenesis of non-obese MAFLD.

These phospholipid species have also been implicated in the development of CVD [174,175]. In this review study, an inverse correlation between PC and/or lyso-PC levels and CVD risk factors was found by Paapstel et al. in AS patients [27], by Chevli et al. in patients with T2D [21], and by Gadgil et al. in individuals with an unhealthy diet [24] (Table 2, Figure 3). Notably, in the study by Paapstel et al. [27], decreased serum levels of many of these lipid species were associated with either increased arterial stiffness, increased resting heart rate, or poorer endothelial function in patients with symptomatic AS, such as patients with CAD and patients with PAD. On the other hand, Syme et al. [28] found that in adolescents, PC16:0/2:0 was negatively associated with CVD risk factors, whereas lysoPC PC14:1/0:0 was positively associated (Table 2, Figure 3). PCs and lysoPCs are important members of the glycerophospholipid family, which play an indispensable structural role in all cell membranes, blood lipoproteins, natural surfactants, and bile, to name but a few. Partial hydrolysis of a PC molecule by phospholipase A2 (PLA2) or PLA1 leads to the formation of lysoPC [176,177], a bioactive lipid involved in monocyte recruitment, vascular smooth muscle cell proliferation, and endothelial cell dysfunction [178]. Recently, Dzobo et al. [179] identified a key role for the lyso-PC derivative lysophosphatidic acid (LPA) and diacylglycerols (DGs) in the monocyte inflammatory response triggered by lipoprotein(a) (Lp(a)). Several studies have shown that Lp(a) can be considered an independent and likely causal risk factor for CVD [180,181,182]. Consistent with these findings, Dzobo et al. [179] found that the LPA precursor lysoPC and the product of LPA degradation, DG [183,184], were enriched in the Lp(a) fraction of individuals with elevated Lp(a) levels compared to healthy individuals with low/normal Lp(a) levels. Functional studies have shown that DGs and LPA play a potential role in Lp(a)-induced monocyte inflammation by promoting cytokine secretion and trans-endothelial migration of monocytes [179], two well-known AS signatures. The evidence provided here is consistent with the findings of Syme et al. [28] of increased lysoPC levels (PC 14:1/0:0) in adolescents with CVD risk factors, such as excess visceral fat, elevated blood pressure, insulin resistance, and atherogenic dyslipidemia. On the other hand, the low PC 16:0/2:0 levels in the same population suggest that the action of the PL regarding CV risk may be type-specific. Based on the findings of Dzobo et al. [179], the lower levels of some PCs and/or lysoPC found by Paapstel et al., Gadgil et al., and Syme et al. [24,27,28] in subjects at CVD risk could be explained by their conversion to LPA. Alternatively, it has been previously hypothesized that the lower serum levels of lyso-PC in patients with CHD may be due to their more efficient removal from the blood into tissues, either in the form of Ox-LDL or directly from albumin [185], which is the major form of plasma LPC [186]. Furthermore, reduced PC concentrations in genetically obese Berlin Fat Mouse Inbred (BFMI mice), an animal model of metabolic syndrome, have been suggested to depend on increased adipocyte turnover, which requires PC as an essential membrane component [187].

#### 4.3.2. FAs

FAs have a decisive impact on health, including the development of AS and CV risk. FAs themselves and their derivatives are known as mediators of both vascular inflammation and resolution of inflammation [188]. These ambivalent biological functions of FAs depend on their quality, quantity, and the availability of enzymes responsible for their metabolism in the human body [189]. The simplest classification divides FAs into the group of SFAs, which have no double bond, and the group of UFAs, which have at least one double bond. Although SFAs are essential for human life [190], studies have shown that excessive consumption of SFAs leads to increased LDL-C concentrations [191], a known causal factor for atherosclerotic CVD [192]. Accordingly, the study by Chen et al. [30] discussed here found that SFAs, particularly long-chain SFA palmitate (PA), were significantly elevated in the plasma of patients with stable AS compared to healthy subjects (Table 2, Figure 3). This finding suggests that AS development may lead to an overproduction of palmitate. Palmitates are the salts and esters of palmitic acid (PA). PA, the most abundant free FA in humans, is an important AS risk factor, as it can induce physiological dysfunction in ECs [193]. Interestingly, in a recent study by Li et al. [194], PA was found to stimulate ECs to autonomously increase the expression of EC-specific molecule 1 (ESM1), a marker of activated ECs [195], which, in turn, may counteract the deleterious effects of PA on ECs. On the other hand, the study by Christensen et al. [29] analyzed here found low SFA levels in children with FH not treated with statins who showed a pro-atherogenic lipid profile (Table 2, Figure 3). According to the authors’ discussion, the lower content of SFAs in the diet of FH children compared to that of healthy controls [196] could justify the observed difference with regard to plasma FA composition.

Short-chain FAs (SCFAs), such as butyrate, propionate, and acetate, are a class of SFAs known to regulate immune responses and inflammation, thereby influencing angiogenesis. Recently, Wada et al. [197] have shown that oral administration of acetate suppresses the formation and progression of AS in ApoE^−/−^ mice by inhibiting macrophage activity, thereby ameliorating plaque formation and progression. Accordingly, two studies reported here found that acetate concentrations were reduced in individuals at high CV risk, such as FH children [29] and DS patients [11] (Table 2, Figure 3).

UFAs are generally divided into monounsaturated FAs (MUFAs) with only one double bond and PUFAs with two or more double bonds. PUFAs are important components of cell membranes, and changes in their relative proportions can affect cell function by modulating the fluidity of membranes [198] and by altering the synthesis of lipid second messengers, including eicosanoids [199]. Some pro-inflammatory eicosanoids may be involved in the development of AS and CVD, whereas others have anti-inflammatory effects and are important factors in CVD prevention. In particular, arachidonic acid (AA), eicosapentaenoic acid (EPA), and docosahexaenoic acid (DHA) are precursors of both pro-inflammatory and anti-inflammatory eicosanoids [188], so an imbalance between their formation may favor CVD development [200]. Due to the ambivalent biological functions of PUFAs and their derivatives, contradictory results were reported in the studies analyzed in this systematic review. The study by Menaker et al. [18] showed that reduced PUFA–eicosadienoic acid levels were associated with AS and a high risk of CVD in obese individuals (Table 2, Figure 3). In addition, Chevli et al. [21] and Zheng et al. [16] found decreased PUFA levels in T2D patients and individuals on SRFB diets, respectively (Table 2, Figure 3). Sugar-sweetened beverages have been consistently reported to be inversely associated with HDL-cholesterol levels [201] and positively associated with LDLs and triglycerides (TGs) [202], indicating the potential contribution of high-added-sugar foods to CVD risk. In addition, a recent study [203] found that higher consumption of PUFA-rich foods was associated with a lower risk of vulnerable intraplaque hemorrhage (IPH) plaques in individuals with subclinical CAS, suggesting that dietary FAs may be a modifiable risk factor for altering carotid artery plaque vulnerability. Consistent with this, Gadgil et al. [23] found increased levels of PUFAs in AS patients without CVD who had a healthy diet. On the other hand, the study by Christensen et al. [29] showed that the levels of some PUFA were elevated in FH children compared to healthy children (Table 2, Figure 3). As mentioned above, this could be related to the different fat dietary intake in the two groups, which could explain the difference in FA plasma composition [29].

#### 4.3.3. Ketone Bodies

3-HB is a ketone body formed by the *β*-oxidation of FAs in the liver and transported to extrahepatic tissues to serve as an energy source [204]. Previous studies have shown that 3-HB has several potential benefits in the treatment of CVD [205,206]. In addition, recently, Zhang et al. [207] have found that daily oral administration of 3-HB significantly ameliorated AS in ApoE^−/−^ mice by reducing M1 macrophages and promoting cholesterol efflux via activation of the macrophage G-protein-coupled receptor 109a (Gpr109a) [208]. Consistent with its CVD protective effect, reduced plasma levels of 3-HB were found in smokers by Harada et al. [17] and in patients with altered cIMT-CAC by Tzoulaky et al. [13] (Table 2, Figure 3).

### 4.4. Other Metabolites

#### 4.4.1. Sex Steroids

Three of the studies analyzed here investigated the correlation between androgenic steroids, diet, and CVD risk. In the study by Zheng et al. [16] and Chevli et al. [21], a high-sugar SRFB diet and T2DM, respectively, were found to be associated with reduced levels of androgenic steroids (Table 2, Figure 3). This finding is consistent with feeding studies showing that glucose intake in men leads to a reduction in testosterone levels [209] and that serum testosterone levels are higher in the fasting state [210]. In addition, it has been shown that men with functional hypogonadism and low serum testosterone levels (<16 nmol/L) were more likely to develop T2DM, which is characterized by increased glucose synthesis in the liver and decreased insulin sensitivity [211,212]. Decreased androgenic steroids levels were also found by Menaker et al. [18] in patients with increased BMI and high CVD risk (Table 2, Figure 3). Recently, Guo et al. [213] found that TGs, glucose, and waist-to-height ratio had the best performance in predicting testosterone deficiency in obese men. Since obesity and fat accumulation are closely associated with T2DM, all this evidence supports the strong correlation between glucose levels and androgen levels. As for the mechanism associated with the inhibitory effect of glucose on sex steroid levels, it should be noted that factors that adversely affect the health of the hypothalamic–pituitary–gonadal axis (HPGA) include increased ROS production [214], which results in hyperglycemia [215], among others. Despite the contradictory results in the past [216], several recent studies have demonstrated an inverse correlation between testosterone levels and CVD risk factors. For example, Li et al. found that low testosterone levels are associated with increased atherogenic index of plasma (AIP), a validate biomarker for AS and CVD [217], while Jiang et al. [218] demonstrated the association between low serum testosterone levels and all-cause mortality in male and female patients with CVD. In addition, a positive correlation between erectile dysfunction, which could be associated with testosterone deficiency [219,220], and the incidence and severity of CVD has been recently found by An et al. [221], with common mechanisms including endothelial dysfunction, oxidative stress, and systemic inflammation. In middle-aged men, low testosterone levels have also been associated with obesity-related hypertension, a known CVD risk factor [222]. Furthermore, Li et al. [223] reported that elevated testosterone and sex-hormone-binding globulin levels, earlier onset of puberty, and vertex baldness were associated with increased CVD risk in men. In line, Gagliano-Juca et al. [224] have reported that testosterone replacement therapy increased circulating neutrophils and monocytes in men with hypogonadism and either pre-existing CVD or increased CVD risk, which was associated with an increased risk of venous thromboembolism. Overall, these results emphasize the importance of sex steroids in CV risk stratification and point to potential targets for future research in CVD prevention.

#### 4.4.2. BAs

Elevated BA concentrations were found in T2DM patients with altered c-IMT [31] or CAC [21] (Table 2, Figure 3). In particular, secondary BA metabolism was positively associated with both higher CAC [21] and altered c-IMT [31] in T2DM patients. In particular, Su et al. [31] found that in T2DM patients with altered c-IMT, increased levels of secondary BAs (DCA, and the DCA derivative TDCA) were associated with decreased levels of primary BAs (TCA). The decreased serum TCA levels of the altered C-IMT group might be due to the activation of FXR by elevated DCA levels, which is known to inhibit intestinal cholesterol absorption by inhibiting cholic acid or TCA synthesis [225]. Since the conversion of primary (TCA) to secondary BAs (DCA and TCDA) is due to the action of bacterial 7α-dehydroxylases [226], the increase in the secondary-to-primary BA ratio in T2DM patients with altered c-IMT has suggested a possible role of the GM in AS pathogenesis in diabetic patients. Consistent with these findings, Li et al. [227] recently found that Xiexin Tang, a classical Chinese medicinal formula, can inhibit the overactivation of FXR in AS mice by inhibiting the synthesis of Bas, which act as FXR agonists, and increasing the synthesis of BAs that antagonize FXR activation, such as TCA [225]. Several studies have attempted to clarify the role of GM-derived metabolites in CVD. In particular, various microbial metabolites, together with stress signals and components of the immune system, have been shown to create a complex regulatory axis between cardiac mitochondria and GM that significantly influences CV health [228]. In this context, secondary BAs produced by the GM have a significant impact on mitochondrial function and overall cardiac health [229] by influencing mitochondrial membrane potential, oxidative phosphorylation, and ROS production through interactions with nuclear receptors, such as FXR and Takeda G protein-coupled receptor 5 (TGR5) [230,231]. Although some studies have shown that oral administration of BAs or mimetics improves diabetes [232], hepatic steatosis [233], and colorectal cancer [234] through mechanisms involving the gastrointestinal system, patients with cardiometabolic disease (CMD3) have elevated systemic BAs in the blood [235,236,237], and such circulating BA levels correlate with coronary stenosis or liver inflammation. In addition, Bae et al. [238] recently found that both a Western diet and LDLR deficiency, which mimics human CMD, trigger the release of BAs by extrahepatic organs in pigs, likely contributing to abnormally elevated circulating BA levels and subsequent vascular inflammation and AS development. These studies support the harmful effects of circulating BAs. In line with this idea, the data from Bae et al. [238], Chevly et al. [21], and Su et al. [31] point to the pro-inflammatory effect of circulating BAs on the vascular system. It is noteworthy that the data from Bae’s study suggest that the triggering of BA levels in organs may depend on reduced levels of circulating fibroblast growth factor 19 (FGF19). Consistent with this, reduced circulating FGF19 levels have been demonstrated in CMD patients [239,240], and systemic FGF19 levels inversely correlate with the atherogenic index and other cardiometabolic traits such as insulin resistance [239,240,241,242,243]. Conversely, FGF19 administration improves glucose homeostasis and hepatic steatosis, with recent clinical trials reporting promising results [244,245].

## 5. Limitations

There is considerable heterogeneity among the studies in terms of analytical platforms, type of biospecimens used, fasting status of participants, population characteristics, and statistical adjustments applied. This heterogeneity limits the ability to make a direct comparison between studies. Only a few studies reported external validation or the use of standardized reference materials, which further complicates comparability. Although this review is narrative in nature, publication bias may still influence the reported findings as studies with positive or significant results are more likely to be published than those with negative or null outcomes. Additionally, a formal risk-of-bias assessment was not performed, as the review focused on identifying trends and discussing mechanisms rather than producing pooled effect sizes. Consequently, the findings should be interpreted with caution. Some methodological details, such as the specific parameters used for pathway analysis, remain unclear and require confirmation from the original study authors to ensure accurate interpretations and reproducibility of the findings.

## 6. Conclusions

AS is a condition characterized by a long, initially asymptomatic phase, the progression of which could lead to CVD. Currently, many studies aim to gain a better understanding of the candidate biomarkers associated with CV risk. This seems extremely urgent given the potential application of these biomarkers as therapeutic agents for the diagnosis and treatment of CV disease. In particular, the identification of metabolites associated with subclinical AS (detected by imaging techniques, such as IMT or CAC) as well as established AS may be of great importance in clinical practice, as they are useful for the early diagnosis of CVD risk in individuals who are apparently free of disease or in whom increased CV risk is newly emerging. Overall, the totality of the studies reported here, considered in light of the recent literature, provides a strong rationale for the use of metabolomic analysis to obtain a global profile of circulating metabolites. This approach may help identify a panel of metabolites as candidate biomarkers with improved diagnostic sensitivity and/or specificity compared with currently available markers, potentially enabling early CVD prevention.

## Figures and Tables

**Table 1:** Characteristics of the included studies.

Author	Sample Size and Participant’s Baseline Characteristics	Sample and Method	Results Increased (↑) or Decreased (↓) Candidate Biomarker Concentration Compared to Control Group	Comments
Jury, 2024 [10]	164 females with SLE (14–76 years) divided into 3 age groups: Group 1: (n = 62, ≤25years) Group 2: (n = 50, 26–49 years) Group 3: (n = 52, 50 years) 123 healthy controls (HCs) (13–72 years) divided into 3 age groups: Group 1: (n = 43, ≤25 years) Group 2: (n = 46, 26–49 years) Group 3: (n = 31, 50 years)	Serum ^1^H-NMR	Metabolites dysregulated in SLE across all three age groups HDLs↓ HDL-apolipoprotein (Apo)A1↓ GlycA↑ Metabolites correlated with age in SLE Acetone↑, Citrate↑, Creatinine↑, Glycerol↑, Lactate↑, Pyruvate↑	The metabolic profile of SLE patients of all ages was characterized by decreased HDL subsets, HDL-(Apo)A1, and increased GlycA. In addition, ApoA1 and GlycA were differentially associated with disease activity and serological measures as well as with AS incidence and myocardial infarction mortality risk. Metabolites of the glycolytic pathway increased significantly with age in SLE, were significantly affected by pharmacological treatment, and were associated with T1D and T2D
Hetman, 2023 [11]	42 individuals with DS (14.17 ± 6.7 years) 20 healthy siblings (15.92 ± 8.58 years)	Serum ^1^H-NMR	Acetate↓, Creatinine↓, Formate↓, Glutamine↑, Lysine↑, Proline↓, Pyroglutamate↓, Xanthine↓	People with DS have a pronounced risk of CVD, with reduced HDL and increased LDL levels. Combined with metabolomic differences and a higher BMI and TMI, this indicates an increased risk of AS compared to controls.
Buszewska-Forajta, 2019 [12]	30 women with PCOS (27.9–31.4 years) 30 healthy age- and BMI-matched controls (26.3–31.3 years)	Serum LC-MS GC-MS	Threonine↑, Tryptophan↑ Tyrosine↑ Isoleucine↑ Leucine↑ Phenylalanine↑, Glycine↑ Homocysteine↓ lysoPC 18:2↑, Lactate↑ Uric acid↑ Dehydroepiandrosterone sulfate (DEHA-S)↑, Sphinganine↑ Cholesterol↓	Compared to healthy controls, PCOS women had increased serum levels of PLs, AACs, organic acids, hormones, and sphinganine and decreased total cholesterol. Among the compounds identified, total cholesterol, phenylalanine and DEHA-S, uric acid, and lactic acid were those with the strongest discriminating power.
Tzoulaki, 2019 [13]	3867 participants from MESA free of known CVD at baseline (62.9 ± 10.3 years) 1917 participants from the Rotterdam Study (70.8 ± 5.7 years) 1652 participants from the LOLIPOP Study (54.8 ± 10 years)	Serum ^1^H NMR	5-Oxoproline↓, Alanine↑, Albumin↓, Aspartate↓, Glutamate↓, Glutamine↓, Glycine↑, Histidine↓, Lysine↓, N,N-Dimethylglycine↓, Phenylalanine↓, Tyrosine↓, Glucose↑, Mannose↑, 1,5-AS↑, Creatine↓, Creatinine↓, 3-HB↓, TGs↑, LDL↑, VLDL↑, Cholesterol↑, ApoB↑, Acetaminophen-glucuronide↑, Citrate↓, Lactate↑	AS was assessed by CAC and IMT evaluation. Metabolites associated with AS were largely consistent between coronary and carotid arteries and mainly labelled metabolic pathways overlapping with known CV risk factors. These metabolites revealed disturbances in lipid and carbohydrate metabolism, BCAA and AAC metabolism, oxidative stress, and inflammatory pathways. Analyses of incident CVD events showed inverse associations with creatine, creatinine, and phenylalanine and direct associations with mannose, acetaminophen glucuronide, and lactate as well as ApoB
Vallejo, 2009 [14]	9 patients with NSTEACS (58–84 years) 10 patients with stable AS (58–74 years) 10 healthy subjects (63–64 years)	Plasma GC-MS	In NSTEACS patients vs. healthy controls: Citric acid↓ 4-Hydroxyproline↓ Aspartic acid↓ Fructose↓ Lactate↑ Urea↑ Glucose↑ Valine↑	Citric acid, 4-hydroxyproline, aspartic acid, and fructose were decreased, and lactate, urea, glucose, and valine were increased in NSTEACS patients vs. healthy controls. The decrease in plasma hydroxyproline levels observed in NSTEACS patients may reflect a status of low collagen synthesis and turnover.
Omori, 2020 [15]	176 T2DM Japanese patients who never experienced a CVD (58.4 ± 12.3 years) 40 T2DM patients who survived CAD (66.5 ± 5.4 years)	Plasma GC-MS	Metabolite associated with FMD in non-CVD patients: 3-Aminoisobutyric acid↓, Galactose + Glucose↓, Glucunoic acid↓, Glucose↓, IS↓, Inositol↓, Mannose↓, O-Phosphoethanolamine↓ Metabolite associated to max-IMT in non-CVD patients: 1,5-AS↑, IS↑, Inositol↑, Meso-erythritol↑, Pyroglutamic acid↑, Urea↑	Inositol and IS were significantly associated with max-IMT and FMD in T2DM patients. These metabolites were also significantly associated with CAD. Furthermore, the association between inositol and CAD persisted after adjustment for traditional coronary risk factors.
Zheng, 2014 [16]	1.977 African American participants from the ARIC Study, which were divided in food groups according to dietary intake (mean 53 years)	Serum GC-MS LC-MS	In SRFB group: Creatinine↓ γ-Glutamyl dipeptides↑ 2-HB↓ PUFA↓ 4-Androsten-3β,17β-diol disulfate↓ 5α-Androstan-3β,17β-diol disulfate↓	The relationship between dietary intake and untargeted serum metabolomics revealed that the SRFB food group was associated with more metabolites than the other food groups/categories, and some of these associations may be due to oxidative stress mechanisms.
Harada, 2024 [17]	Study 1: 9922 Japanese participants from the baseline survey TMCS conducted from fiscal year (April-March) 2012–2015, including 4576 men (1335 current cigarette smokers; 2287 past smokers; 954 never smokers) and 5346 women (238 current cigarette smokers; 471 past smokers; 4367 never smokers (35–74 years) Study 2: 3334 participants involved in a follow-up survey of the TMCS conducted from fiscal year 2018–2021, including 55 HTP users (49.22 ±10.57 years), 119 cigarette smokers (54.51 ± 8.83 years), 144 past smokers (52.72 ± 10.25 years), and 145 never smokers (51.34 ± 10.08 years)	Plasma CE-MS/MS	Trends in cigarette smokers Glutamate↑, Proline↑, Tyrosine↑, Ornithine↑, Arginine↑, Citrulline↑, N,N-Dimethylglycine↑, Serine↑, Threonine↑, Leucine↑, Isoleucine↑, Kynurenine↓ Citrates↓ 2-HB↓ 3-HB↓, 2-oxobutyrate↓, Pyruvate↑, Trigonellina↑ Guanidinosuccinate↓, Mucate↓, Uridine↓ Trends in HTP users Glutamate↑, Proline↓, Tyrosine↓, Ornithine↑, Arginine↑, Citrulline↑, N,N-Dimethylglycine↑, Serine↓, Threonine↑, Leucine↓, Isoleucine↓, Kynurenine↓ Citrates↓, 2-HB↓ 3-HB↓, 2-oxobutyrate↑, Piruvate↑, Trigonellina↑ Guanidinosuccinate↓, Mucate↓, Uridine↓	Cigarette smokers had different metabolomic profiles than non-smokers. The metabolite profiles of HTP users were closer to those of cigarette smokers than to those of never smokers. Specifically, cigarette smokers and HTP users had higher concentrations of trigonelline and amino acid metabolites, including glutamate, ornithine, and arginine, than never smokers and past smokers. Metabolites involved in glutamate metabolism (arginine, ornithine, and citrulline) were also associated with cigarette smoking and HTP use.
Menaker, 2024 [18]	216 adults from the 300-OB cohort: (BMI ≥ 27 kg/m^2^) (55–80 years), including the following: (1) women with MetS (W/MetS), with or without AS (healthy subjects); (2) women without MetS (W/NoMetS), with or without AS; (3) men with MetS (M/MetS), with or without AS; and (4) men without MetS (M/NoMetS), with or without AS. Validation cohorts: 473 (202 men and 271 women) healthy subjects from the 500-FG cohort, most with a BMI in the normal range (18–75 years) 2645 healthy middle-aged women from the TwinsUK cohort with an average BMI 30.8 ± 3.6 (average age 59 years) including 49 with at least one CVD event between 4–13 years after data collection	Plasma LC-MS	Metabolite associated with atherogenic state: γ-Glu-Glu↑, NAM↑, HVA↑; HVA sulphate↑ Phenylalanine and tyrosine catabolism↑: Phenylacetylglutamine↑, PAGln acid↑ Phenylpropanoids biosynthesis↑: Ferulic acid↑, Sinapate↑, Kaempferide↑ FAs↓: (including eicosadienoic acid↓,dodecanoic acid↓) Estrogens biosynthesis↓ (including androstenedione↓, estriol↓, pregnenolone↓)	The analysis of groups of individuals with different clinical conditions allowed for the identification of metabolites associated with the atherogenic state independently of the particular condition, such as γ-Glu-Glu and HVA sulfate. Metabolic pathways such as the catabolism of phenylalanine and tyrosine and the biosynthesis of oestrogens and phenylpropanoid were also associated with the atherogenic state. Validation cohorts confirmed the variation in atherogenic states in healthy subjects (before atherosclerotic plaques become visible) and showed that metabolites associated with the atherogenic state were also associated with future CVD.
Shao, 2023 [19]	120 MAFLD patients including the groups non-obese MAFLD with (n = 19, 42.4 ± 11.7 years)/without (n = 41, 43.6 ± 11.7 years) CAS and obese MAFLD with (n = 20, 44.1 ± 12.1 years)/without (n = 40, 45.2 ± 10.8 years) CAS 60 non-MAFLD controls with (n = 30, 44.5 ± 9.9 years)/without (n = 30, 44.6 ± 11.9 years) CAS	Plasma UHPLC-QTOF-MS GC-MS	Metabolite associated with CAS in MAFLD patients Alpha-tocopherol↓, PE (20:2/16:0)↓, L-glutamine↓, PC (18:2/20:2)↓, SM (16:1/18:1)↓, D-threitol↑, PG (18:0/20:4)↓, De novo lipogenesis (DNL) (16:0/18:2n-6)↑, L-leucine↑, Cystine↑	The combination of PE (20:2/16:0), DNL (16:0/18:2n-6), PG (18:0/20:4), and liver stiffness were a strong predictor of CAS in non-obese MAFLD patients. The combination of cystine, SM (16:1/18:1), DNL (16:0/18:2n-6), age, and liver fat content (LFC) correlated with CAS in obese MAFLD patients.
Santiago-Hernandez, 2021 [20]	27 patients with a diagnosis of CAD undergoing CABG (68 ± 9 years) 24 healthy subjects (Age ND)	Plasma Urine LC-MS	Arabitol (urine)↑, Spermidine (urine)↑, Glutamine (urine and plasma)↓, Trimethylamine N-oxide (urine and plasma)↑, Pantothenate (urine)↓, Valine (plasma)↓, Acetylcholine (plasma)↓, Choline (plasma)↓, Pyruvate (plasma)↑	The observed metabolic deregulations in CAD patients undergoing CABG indicated an inflammatory response together with a altered counteraction of oxidative stress
Chevli, 2021 [21]	700 participants from DHS: 438 AAs (58.7 ± 8.8 years) and 262 EAs (61.8 ± 9 years) Unaffected family members	Plasma LC-MS	AAs: Androgenic steroids↓, BAs↑, PCs↓, γ-GPs↓, Acylcarnitines↓, Dicarboxilate FAs↓, Monohydroxy FAs↓, Medium-chain FAs↓, Pregnenolone steroids↓ EAs: Androgenic steroids↓, BCAA metabolites↑, N-acetylphenylalanine↑, Lysine metabolites↑, Lysoplasmalogens↑, Pregnenolon/progestin steroids↓, Campesterol↑, 3-Hydroxy-3-methylglutarate↑	Androgenic steroid, FAs, and BA metabolism subpathways were significantly associated with CAC in AAs, whereas androgenic steroid, progestin steroid, pregnenolone steroid, lysoplasmalogen, sphingomyelin, and BCAA metabolism subpathways were associated with CAC in EAs.
Wolak-Dinsmore, 2018 [22]	1209 participants from IRAS: 376 with T2DM (57 ± 8 years) and 833 non-diabetic (ND) subjects (55 ± 8 years) 123 participants from the Groningen cohort: 67 T2D patients (59 ± 9 years) and 56 ND (54 ± 10 years)	Plasma NMR LC-MS	Isoleucine↑ Leucine↑ Valine↑	BCAA levels were elevated in T2DM patients and were associated with c-IMT, an indicator of subclinical AS.
Gadgil, 2023 [23]	3557 participants without known CVD from MESA cohort (45–84 years)	Serum ^1^H NMR	Metabolites associated to a higher HEI 2015 score: Proline↓, Proline betaine↑,1,5 AS↓, C=CHCH2HC=C (fatty acyl chains) UFA↑	Diet quality, measured by HEI-2015, was positively associated with proline betaine and inversely related to proline, 1,5-AS, and UFA chains.
Gadgil, 2022 [24]	722 participants from the MASALA cohort study without known CVD (40–84 years) were categorized into three dietary patterns based on their eating habits: “Fruits, Vegetables, Nuts, Legumes” pattern, “Animal Protein” pattern, and “Fried Snacks, Sweets, High-Fat Dairy” pattern	Serum UPLC-MS	“Animal Protein” diet: PE (O-16:1/20:4) and/or PE(P-16:0/20:4)↑, NAPE (O-18:1/20:4/18:0) and/or NAPE (P-18:0/20:4/18)↑, LPI (22:6/0:0)↑, FA (22:6)↑ “Fried Snacks, Sweets, High-Fat Dairy” diet: PC (16:0/22:6)↓, FA (22:6)↓ “Fruits, Vegetables, Nuts, Legumes” diet: Proline betaine↑	The pattern “Fruits, Vegetables, Nuts, Legumes” was positively associated with proline betaine and was linked to a lower risk of diabetes. The pattern “Animal Protein” was associated with NAPEs, sphingomyelins, and ceramides as well as long- and short-chain acylcarnitines. In addition, the patterns “Animal Protein” and “Fried Snacks, Sweets, High-Fat Dairy” showed contrasting associations with long-chain n–3 FAs, which were associated with a lower CVD risk.
Benitez, 2022 [25]	325 samples from patients with moderate-to-severe CKD and a median follow-up of 2 years, including 149 patients from the RRI-CKD study and 199 newly recruited patients, divided as follows: patients with no history of CVD (n = 198) (57 ± 14.4 years) patients with a history of CVD (n = 127) (65.5 ± 13.9 years) patients who experienced new CV events during the study period (n = 50) (66.9 ± 12.3 years) patients who had no new CV events during the study period (n = 275) (59.2 ± 14.9 years)	Plasma LC-MS	Tryptophan↓, Hydroxyanthranilic acid↑, Quinolinic acid↑, Kynurenine↑	The kynurenine pathway, but not indole metabolites, plays a role in subclinical AS and new CV events in advanced CKD. These data support a possible role of altered tryptophan immunometabolism in the pathogenesis of CKD-associated AS.
Ho, 2022 [26]	119 individuals with PAD with ABI < 0.9 (65–73 years) 37 controls without apparent AS (60–74 years)	Plasma LC-MS	Kynurenine↓, Tryptophan↓, HA↓, IPA↓, I3A↓, IS↑, HAA↑	This study investigates the association of microbiota-derived metabolites with PAD and MACE. After adjustment for traditional AS risk factors, concentrations of kynurenine, HA, IPA, and I3A were negatively associated with PAD, while IS and HAA were positively associated. HA, IPA, and I3A correlated with the ABI. Participants in the quartile with the highest I3A concentration had significantly higher freedom from MACE during the follow-up period than those in the lowest quartile
Paapstel, 2018 [27]	32 males with PAD (61.7 ± 9.0 years) 52 males with CAD (63.2 ± 9.2 years) 40 healthy controls (60.3 ± 7.1 years)	Serum LC-MS	PC aa 28:1↓ PC aa 30:0↓ PC aa 32:2↓ PC ae 30:0↓ PC ae C34:2↓ lysoPC a 18:2↓	Changes in the PC and lysoPC profiles were observed in all three study groups. The lower serum levels of many of these lipids were associated with either increased arterial stiffness, increased resting HR, or poorer endothelial function in AS patients. Despite some similarities, patients with PAD and CAD may differ in the way their lipid profiles relate to other biochemical and functional parameters.
Syme, 2016 [28]	990 adolescents (12–18 years, 48% male) as part of the SYS	Serum LC-MS	GPCs: PC 14:1/0:0↑ PC 16:0/2:0↓	Targeted serum lipidomics was used to identify GPCs associated with CV risk factors (such as VF, BP, TGs, and HDL-cholesterol) in adolescents. Most significantly, PC16:0/2:0 was negatively associated with CVD risk factors, while a direct association was observed for PC14:1/0:0.
Christensen, 2017 [29]	47 children with FH (12.5 ± 3.7 years) 57 healthy children (10 ± 1.9 years)	Plasma ^1^H NMR	Acetate↓ Acetoacetate↓ PUFA↑: DHA↑, FAω3↑, FAω6↑, Linoleic acid↑, MUFA↑, SFAs↓ Cholesterol↑, LDL↑, VLDL↑, ApoB↑, XL-HDL↑, M-HDL↓, S-HDL↓	FH children had higher levels of ApoB-containing lipoproteins and lipids as well as lipid fractions in lipoprotein subclasses compared to healthy children (HC). In addition, they showed changes in HDL particle concentration and lipid content compared to HC. In addition, non-statin-treated FH children had higher plasma FAs than HC, especially linoleic acid. Finally, acetoacetate and acetate were lower in FH children compared to HC.
Chen, 2010 [30]	16 patients with stable AS 28 age- and sex-matched healthy subjects (Age ND)	Plasma GC-MS	Stearate↑ Palmitate↑ 1-Monolinoleoylglycerol↑	AS development directly affects FA metabolism, particularly that of palmitate, which has been confirmed as a phenotypic candidate biomarker for the clinical diagnosis of AS.
Su, 2021 [31]	231 T2DM patients with altered c-IMT (55–66 years) 231 T2DM patients with normal c-IMT (54–67 years)	Serum LC-MS	DCA↑, TDCA↑, TCA↓	Serum bile acid levels were independently associated with c-IMT, suggesting that BAs may contribute to the development of ASCVD.
Wang, 2023 [32]	433 women with or at high risk of HIV (65% HIV +) from the Women’s Interagency HIV Study (WIHS) 320 women from WIHS who underwent carotid artery imaging (46–62 years)	Plasma LC-MS	Metabolites associated with CAC ImP↑, 3-Hydroxyhippuric acid↑	Altered gut microbial species, serum inflammatory markers, and plasma metabolites were associated with CAC in women with or at risk of HIV. Among the plasma metabolites associated with plaque-associated microbial species, 3-hydroxyhippuric acid, and ImP were positively associated with plaque and several pro-inflammatory markers. However, further analyses suggested that the associations between the identified gut bacterial species and plaque in the carotid artery were independent of circulating 3-hydroxyhippuric acid levels but dependent on ImP plasma levels.
Akawi, 2021 [33]	Study 1: 633 AS patients undergoing CABG recruited under the ox-HVF cohort (66.6 ± 9.7 years) Study 2: a subgroup of 48 patients of Study 1, including 31 obese (62.1 ± 11.9 years) and 17 lean (63.4 ± 8.4 years) subjects Study 3: 32 healthy obese subjects (51.4 ± 9.9 years)	Plasma LC-MS	C16:0-Ceramide↑ C16:0-Glycosylceramide↑	Circulating C16:0 ceramide correlated positively with thoracic adipose tissue ceramides, dysregulated vascular redox signalling, and increased systemic inflammation in AS patients. High plasma C16:0 ceramide and its glycosylated derivative were independently associated with an increased risk of cardiac mortality in advanced AS.

AS: atherosclerosis; NSTEACS: non-ST elevation acute coronary syndrome; ARIC: Atherosclerosis Risk in Communities; VF: visceral fat; BP; blood pressure; CVD: cardiovascular disease; FA: fatty acid; PUFA: polyunsaturated fatty acids; SRFB: sugar rich food and beverages; SYS: Saguenay Youth Study; 1,5 AS: 1,5-anhydrosorbitol; GPCs: glycerophosphocholines; PC: phosphocholine; TGs: triglycerides; HDL: high-density lipoprotein; CV: cardiovascular; FH: familial hypercholesterolemia; DHA: docosahexaenoic acid; BAs: bile acids; FAω3: Omega 3 FAs; FAω6: Omega 6 FAs; MUFA: monounsaturated fatty acid; SFAs: saturated fatty acids: LDL: low-density lipoproteins; VLDL: very low-density lipoproteins; ApoB: apolipoprotein B; XL-HDL: extra-large HDL; M-HDL: medium HDL; S-HDL: small HDL; PAD: peripheral arterial disease; CAD: coronary artery disease; lysoPC: lysophosphatidylcholine; HR: heart rate; IRAS: Insulin Resistance Atherosclerosis Study; T2DM: type 2 diabetes mellitus; BCAA: branched-chain amino acid; IMT: intima-media thickness; c-IMT: carotid intima-media thickness; PCOS: polycystic ovary syndrome; AAC: aromatic amino acid; BMI: body mass index; DEHA-S: dehydroepiandrosterone sulfate; 2-HB: 2-hydroxybutirate; 3-HB: 3-hydroxybutyrate; γ-GPs: γ-glutamylpeptides; ImP: imidazolyl propionate; MESA: Multi-Ethnic Study of Atherosclerosis; LOLIPOP: London Life Sciences Prospective Population Study; CAC: coronary artery calcification; max-IMT: maximal intima-media thickness; FMD: flow-mediated vasodilation; DCA: deoxycholic acid; TDCA: tauro deoxycholic acid; TCA: taurocholic acid; ASCVD: atherosclerotic cardiovascular disease; CABG: coronary artery bypass grafting; ox-HVF: Oxford Heart Vessels and Fat; DHS: Diabetes Heart Study; AAs: African Americans; EAs: European Americans; CKD: chronic kidney disease; RRI-CKD: Renal Research Institute Chronic Kidney Disease; ABI: ankle–brachial index; IS: indoxyl sulfate; HA: hippuric acid; IPA: indole-3-propionic acid; I3A: indole-3-aldehyde; HAA: 3-hydroxyanthranilic acid; MACE: major adverse cardiac events; MASALA: Mediators of Atherosclerosis in South Asian living in America; PE: phosphatidylethanolamine; NAPE: N-acyl phosphatidylethanolamine; LPI: lysophosphatidylinositol; MAFLD: metabolic-disfunction-associated fatty liver disease; CAS: carotid atherosclerosis; SM: sphyngomyelin; PG: phosphatidylglycerol; DNL: de novo lipogenesis; WIHS: Women’s Interagency HIV Study; HIV: human immunodeficiency virus; ImP: imidazolyl propionate; HEI: Healthy Eating Index; UFA: unsaturated fatty acid; TMCS: Tsuruoka Metabolomics Cohort Study; HTP: heated tobacco products; DS: Down’s syndrome; TMI: Tri-Ponderal Mass Index; SLE: systemic lupus erythematosus; GlycA: glycoprotein acetyls; (Apo)A1: apolipoprotein A1; T1D: type 1 diabetes; MetS: metabolic syndrome; γ-Glu-Glu: γ-L-glutamil-L-glutamic acid; NAM: nicotinate D-ribonucleotide; HVA: homovanillic acid; MS: mass spectrometry; GC-MS: gas chromatography–mass spectrometry; LC-MS: liquid chromatography–mass spectrometry; NMR: nuclear magnetic resonance.

**Table 2:** Altered metabolites identified in the included studies.

Metabolite	Metabolite [ ] Trend	Type of Sample	CV Risk Factor/CV Protection Factor *	Study
Metabolism of amino acids and derivatives				
Phenylalanine	↑	Serum	PCOS	Buszewska-Forajta, 2019 [12]
	↓	Serum	Altered CAC-IMT	Tzoulaki, 2019 [13]
N-acetylphenylalanine	↑	Plasma	T2DM	Chevli, 2021 [21]
Phenylacetylglutamine (PAGln)/PAGln acid	↑	Serum	Obesity and atherosclerosis	Menaker, 2024 [18]
Tyrosine	↑	Serum	PCOS	Buszewska-Forajta, 2019 [12]
	↑	Serum	Smoke	Harada, 2024 [17]
	↑	Serum	Obesity and atherosclerosis	Menaker, 2024 [18]
	↓	Serum	Altered CAC-IMT	Tzoulaki, 2019 [13]
Creatinine	↓	Serum	DS	Hetman, 2023 [11]
	↓	Serum	Altered CAC-IMT	Tzoulaki, 2019 [13]
	↓	Serum	Unhealthy diet	Zheng, 2014 [16]
	↑	Serum	LES	Jury, 2024 [10]
Lysine	↓	Serum	Altered CAC-IMT	Tzoulaki, 2019 [13]
	↑	Serum	DS	Hetman, 2023 [11]
Threonine	↑	Serum	PCOS	Buszewska-Forajta, 2019 [12]
	↑	Serum	Smoke	Harada, 2024 [17]
Urea	↑	Plasma	NSTEACS	Vallejo, 2009 [14]
	↑	Plasma	T2DM with altered c-IMT	Omori, 2020 [15]
Glutamate	↑	Plasma	Smoke	Harada, 2023 [17]
	↓	Serum	Altered CAC-IMT.	Tzoulaki, 2019 [13]
γ-L-Glutamil-L-Glutamic acid (γ-Glu-Glu)	↑	Serum	Obesity and atherosclerosis	Menaker, 2024 [18]
Glutamine	↓	Serum	LES	Jury, 2024 [10]
	↓	Serum	Altered CAC-IMT	Tzoulaki, 2019 [13]
	↓	Urine/Plasma	CAD	Santiago-Hernandez, 2021 [20]
	↓	Plasma	MAFLD w/without obesity	Shao, 2023 [19]
	↑	Serum	DS	Hetman et al. [11]
Aspartate	↓	Serum	Altered CAC-IMT	Tzoulaki, 2019 [13]
	↓	Plasma	NSTEACS	Vallejo, 2009 [14]
Branched-chain amino acids (BCAAs)	↑	Plasma	T2DM with altered CAC	Chevli, 2021 [21]
Leucine	↑	Plasma	T2DM with altered c-IMT	Wolak-Dinsmore, 2018 [22]
	↑	Serum	Smoke	Harada, 2024 [17]
	↑	Serum	PCOS	Buszewska-Forajta, 2019 [12]
Isoleucine	↑	Plasma	T2DM with altered c-IMT	Wolak-Dinsmore, 2018 [22]
	↑	Serum	Smoke	Harada, 2024 [17]
	↑	Serum	PCOS	Buszewska-Forajta, 2019 [12]
Valine	↑	Plasma	T2DM with altered c-IMT	Wolak-Dinsmore, 2018 [22]
	↑	Plasma	NSTEACS	Vallejo, 2009 [14]
	↓	Urine/Plasma	CAD	Santiago-Hernandez, 2021 [20]
Proline	↓	Serum	Smoke	Harada, 2024 [17]
	↓	Serum	Healthy diet *	Gadgil, 2023 [23]
	↓	Serum	DS	Hetman, 2023 [11]
Proline betaine	↑	Serum	Healthy diet *	Gadgil, 2022 [24]
	↑	Serum	Healthy diet *	Gadgil, 2023 [23]
Tryptophan	↑	Serum	PCOS	Buszewska-Forajta, 2009 [12]
	↓	Plasma	PAD	Ho, 2023 [26]
	↓	Plasma	CKD	Benitez, 2022 [25]
Kynurenine	↑	Plasma	CKD	Benitez, 2022 [25]
	↓	Serum	Smoke	Harada, 2024 [17]
	↓	Plasma	PAD	Ho, 2023 [26]
3-Hydroxyanthranilic acid (HAA)	↑	Plasma	CKD	Benitez, 2022 [25]
	↑	Plasma	PAD	Ho, 2023 [26]
Indoxyl sulfate (IS)	↑	Plasma	PAD	Ho, 2023 [26]
	↑	Plasma	T2DM with altered c-IMT	Omori, 2020 [15]
γ-Glutamyl dipeptides (γ-GPs)	↑	Serum	SRFB diet	Zheng, 2014 [16]
	↓	Plasma	T2DM with altered CAC	Chevli, 2021 [21]
N,N-Dimethylglycine (DMG)	↑	Serum	Smoke	Harada, 2024 [17]
	↓	Serum	Altered CAC-IMT	Tzoulaki, 2019 [13]
2-Hydroxybutyrate (2-HB)	↑	Serum	SRFB diet	Zheng, 2014 [16]
	↓	Serum	Smoke	Harada, 2024 [17]
3-Hydroxybutyrate (3-HB)	↓	Serum	Smoke	Harada, 2024 [17]
	↓	Serum	Altered CAC-IMT	Tzoulaki, 2019 [13]
Carbohydrate and energy metabolism				
Glucose	↑	Serum	Altered CAC-IMT	Tzoulaki, 2019 [13]
	↑	Serum	NSTEACS	Vallejo, 2009 [14]
Pyruvate	↑	Serum	Smoke	Harada, 2024 [17]
	↑	Serum	PCOS	Buszewska-Forajta, 2009 [12]
	↑	Urine	CAD	Santiago-Hernandez, 2021 [20]
Lactate	↑	Serum	Altered CAC-IMT	Tzoulaki, 2019 [13]
	↑	Serum	NSTEACS	Vallejo, 2009 [14]
	↑	Serum	PCOS	Buszewska-Forajta, 2009 [12]
	↑	Serum	LES	Jury, 2024 [10]
1,5-Anhydro-D-glucitol (1,5 AG)	↑	Plasma	T2DM with altered c-IMT	Omori, 2020 [15]
	↑	Serum	Altered CAC-IMT	Tzoulaki, 2019 [13]
	↓	Serum	Healthy diet *	Gadgil, 2023 [23]
Citrate	↓	Serum	Altered CAC-IMT	Tzoulaki, 2019 [13]
	↓	Serum	Smoke	Harada, 2024 [17]
	↓	Plasma	NSTEACS	Vallejo, 2009 [14]
	↑	Serum	LES	Jury, 2024 [10]
Metabolism of lipids and derivatives				
Phosphatidylethanolamine (PE)				
PE 20:2/16:0	↓	Plasma	MAFLD w/without obesity	Shao, 2023 [19]
PE O-16:1/20:4	↑	Serum	Animal Protein diet	Gadgil, 2022 [24]
PE P-16:0/20:4	↑	Serum	Animal Protein diet	Gadgil, 2022 [24]
N-acyl phosphatidylethanolamine (NAPE)				
NAPE O-18:1/20:4/18:0	↑	Serum	Animal Protein diet	Gadgil, 2022 [24]
NAPE P-18:0/20:4/18	↑	Serum	Animal Protein diet	Gadgil, 2022 [24]
Lysophosphatidylinositol (LPI) 22:6/0:0	↑	Serum	Animal Protein diet	Gadgil, 2022 [24]
Phosphatidylglycerol (PG) (18:0/20:4)	↓	Plasma	MAFLD w/without obesity	Shao, 2023 [19]
Lysophosphatidylcholine (lysoPC) a 18:2	↓	Serum	PAD/CAD	Paapstel, 2018 [27]
lysoPC PC14:1/0:0	↑	Serum	VF, BP, insulin, TGs, and HDL-cholesterol in adolescence	Syme, 2016 [28]
Phosphatidylcholines (PCs)	↓	Plasma	T2DM with altered CAC	Chevli, 2021 [21]
PC (18:2/20:2)	↓	Plasma	MAFLD w/without obesity	Shao, 2023 [19]
PC aa 30:0	↓	Serum	PAD/CAD	Paapstel, 2018 [27]
PC aa 32:2	↓	Serum	PAD/CAD	Paapstel, 2018 [27]
PC ae 30:0	↓	Serum	PAD/CAD	Paapstel, 2018 [27]
PC ae C34:2	↓	Serum	PAD/CAD	Paapstel, 2018 [27]
PC aa 30:0	↓	Serum	PAD/CAD	Paapstel, 2018 [27]
PC aa 32:2	↓	Serum	PAD/CAD	Paapstel, 2018 [27]
PC 16:0/22:6	↓	Serum	Fried Snacks, Sweets, High-Fat Dairy diet	Gadgil, 2022 [24]
PC 16:0/2:0	↓	Serum	VF, BP, insulin, TGs, and HDL-cholesterol in adolescence	Syme, 2016 [28]
Saturated fatty acids (SFAs)	↓	Plasma	FH in children	Christensen, 2017 [29]
Palmitate (PA)	↑	Plasma	Stable atherosclerosis	Chen, 2010 [30]
Acetate	↓	Plasma	FH in children	Christensen, 2017 [29]
	↓	Serum	Smoke	Harada, 2024 [17]
Polyunsaturated fatty acids (PUFA)	↓	Plasma	T2DM with altered CAC	Chevli, 2021 [21]
	↓	Serum	SRFB diet	Zheng, 2014 [16]
	↑	Plasma	FH in children	Christensen, 2017 [29]
Eicosadienoic acid	↓	Serum	Obesity and atherosclerosis	Menaker, 2024 [18]
C=CHCH_2_HC=C (fatty acyl chains)	↑	Serum	Healthy diet *	Gadgil, 2023 [23]
Omega 3 FAs (FAω3),	↑	Plasma	FH in children	Christensen, 2017 [29]
Omega 6 FAs (FAω6)	↑	Plasma	FH in children	Christensen, 2017 [29]
Linoleic acid	↑	Plasma	FH in children	Christensen, 2017 [29]
Monounsaturated FAs (MUFA)	↑	Plasma	FH in children	Christensen, 2017 [29]
3-Hydroxybutyrate (3-HB)	↓ ↓	Serum Serum	Smoke Altered CAC-IMT	Harada, 2024 [17] Tzoulaki, 2019 [13]
De novo lipogenesis (DNL) (16:0/18:2n-6)	↑	Plasma	MAFLD w/without obesity	Shao, 2023 [19]
Other metabolites				
Androgenic steroids	↓	Serum	SRFB diet	Zheng, 2014 [16]
	↓	Plasma	T2DM with altered CAC	Chevli, 2021 [21]
Bile acids (BAs)	↑	Serum	T2DM with altered c-IMT	Su, 2021 [31]
	↑	Serum	T2DM with altered CAC	Chevli, 2021 [21]

Up/down arrows indicate increased/decreased metabolite concentration. * indicates protection factors, such as healthy diet.

## Supplementary Materials

The following supporting information can be downloaded at: https://www.mdpi.com/article/10.3390/jcm14228028/s1, Supplementary File S1: PRISMA 2020 Main Checklist.

## Abbreviations

AS	atherosclerosis
NSTEACS	non-ST elevation acute coronary syndrome
ARIC	Atherosclerosis Risk in Communities
VF	visceral fat
BP	blood pressure
CVD	cardiovascular disease
FA	fatty acid
PUFA	polyunsaturated fatty acids
SRFB	sugar-rich food and beverages
SYS	Saguenay Youth Study
GPC	glycerophosphocholines
PC	phosphocholine
TGs	triglycerides
2-HB	2-hydroxybutirate
3-HB	3-hydroxybutyrate
HDL	high-density lipoprotein
CV	cardiovascular
FH	familial hypercholesterolemia
DHA	docosahexaenoic acid
FAω3	Omega 3 FAs
FAω6	Omega 6 FAs
MUFA	monounsaturated fatty acid
SFAs	saturated fatty acids
LDL	low-density lipoproteins
VLDL	very low-density lipoproteins
ApoB	apolipoprotein B
XL-HDL	extra-large HDL
M-HDL	medium HDL
S-HDL	small HDL
PAD	peripheral arterial disease
CAD	coronary artery disease
lysoPC	lysophosphatidylcholine
HR	heart rate
GM	gut microbiota
SI	sarcopenia index
IRAS	Insulin Resistance Atherosclerosis Study
T2DM	type 2 diabetes mellitus
BCAA	branched-chain amino acid
IMT	intima-media thickness
c-IMT	carotid intima-media thickness
PCOS	polycystic ovary syndrome
AAC	aromatic amino acid
1,5 AS	1,5-anhydrosorbitol
BMI	body mass index
DEHA-S	dehydroepiandrosterone sulphate
MESA	Multi-Ethnic Study of Atherosclerosis
LOLIPOP	London Life Sciences Prospective Population Study
CAC	coronary artery calcification
max-IMT	maximal intima-media thickness
FMD	flow-mediated vasodilation
DCA	deoxycholic acid
TDCA	tauro deoxycholic acid
TCA	taurocholic acid
ASCVD	atherosclerotic cardiovascular disease
CABG	coronary artery bypass grafting
ox-HVF	Oxford Heart Vessels and Fat
DHS	Diabetes Heart Study
AAs	African Americans
EAs	European Americans
CKD	chronic kidney disease
RRI-CKD	Renal Research Institute Chronic Kidney Disease
ABI	ankle–brachial index
HA	hippuric acid
IPA	indole-3-propionic acid
I3A	indole-3-aldehyde
HAA	3-hydroxyanthranilic acid
BHMT	betaine homocysteine methyltransferase
MACE	major adverse cardiac events
MASALA	Mediators of Atherosclerosis in South Asian living in America
PE	phosphatidylethanolamine
NAPE	N-acyl phosphatidylethanolamine
LPI	lysophosphatidylinositol
MAFLD	metabolic-disfunction-associated fatty liver disease
CAS	carotid atherosclerosis
SM	sphingomyelin
PG	phosphatidylglycerol
DNL	de novo lipogenesis
WIHS	Women’s Interagency HIV Study
HIV	human immunodeficiency virus
ImP	Imidazolyl propionate
HEI	Healthy Eating Index
UFA	unsaturated fatty acids
CaSR5	calcium-sensing receptor
TMCS	Tsuruoka Metabolomics Cohort Study
HTP	heated tobacco products
DS	Down’s syndrome
TMI	Tri-Ponderal Mass Index
SLE	systemic lupus erythematosus
GlycA	glycoprotein acetyls
(Apo)A1	apolipoprotein A1
T1D	type 1 diabetes
MetS	metabolic syndrome
γ-Glu-Glu	γ-L-glutamil-L-glutamic acid
NAM	nicotinate D-ribonucleotide
HVA	homovanillic acid
MS	mass spectrometry
GM-MS	gas chromatography–mass spectrometry
LC-MS	liquid chromatography–mass spectrometry
NMR	nuclear magnetic resonance
SMCs	smooth muscle cells
FXR	farnesoid X-activated receptor
CRF	chronic renal failure
